# Tectonic settings influence the geochemical and microbial diversity of Peru hot springs

**DOI:** 10.1038/s43247-023-00787-5

**Published:** 2023-04-11

**Authors:** Heather E. Upin, Dennis L. Newell, Daniel R. Colman, Eric S. Boyd

**Affiliations:** 1 Department of Geosciences, Utah State University, Logan, UT USA; 2 Department of Microbiology and Cell Biology, Montana State University, Bozeman, MT USA

**Keywords:** Biogeochemistry, Geochemistry

## Abstract

Tectonic processes control hot spring temperature and geochemistry, yet how this in turn shapes microbial community composition is poorly understood. Here, we present geochemical and 16 S rRNA gene sequencing data from 14 hot springs from contrasting styles of subduction along a convergent margin in the Peruvian Andes. We find that tectonic influence on hot spring temperature and geochemistry shapes microbial community composition. Hot springs in the flat-slab and back-arc regions of the subduction system had similar pH but differed in geochemistry and microbiology, with significant relationships between microbial community composition, geochemistry, and geologic setting. Flat-slab hot springs were chemically heterogeneous, had modest surface temperatures (up to 45 °C), and were dominated by members of the metabolically diverse phylum Proteobacteria. Whereas, back-arc hot springs were geochemically more homogenous, exhibited high concentrations of dissolved metals and gases, had higher surface temperatures (up to 81 °C), and host thermophilic archaeal and bacterial lineages.

## Abstract (web summary)

Differences in temperature and geochemistry linked to tectonic processes influence the microbial community composition and geochemical cycling of elements in hot springs, according to geochemical and genomic analyses conducted in 14 hot springs in the Peruvian Andes.

## Introduction

Continental arcs host geothermal systems with hot spring emanations governed by plate boundary processes. The tectonic setting influences the input of heat and volatiles into the crust, geothermal fluid–rock interaction, structural controls on groundwater flow paths, and the variable rock types encountered by circulating groundwater^1,2^. Hot spring waters derive from deeply circulated meteoric water and despite long-flow paths and mixing with shallow ground waters carry geochemical tracers from the mantle and crust that are windows to deep-seated geological processes linked to the tectonic setting^3–5^. Although it is known that tectonic processes influence the location, temperature, and geochemical composition of geothermal fluids and related hot spring emanations^6,7^, far less is known about whether such processes shape the diversity of microorganisms that inhabit these springs.

The microbial function and composition of continental hot springs are directly connected to local environmental conditions^8,9^. For example, in circumneutral to alkaline hot springs in Yellowstone National Park (YNP), USA, communities supported by light energy (i.e., photosynthetic) are limited to temperatures <73 °C^10^, but <57 °C in acidic and high-sulfide environments^11,12^. Above these temperature limits, life is sustained by chemical energy generated by dissipating disequilibria in available oxidants and reductants^13,14^. This redox disequilibria is largely sustained by the mixing of reduced subsurface fluids and oxidized meteoric water or by infusion of subsurface fluids with oxidized atmospheric gas^13,14^. At magmatic-hydrothermal systems like YNP, Kamchatka, Russia, and Taupo, New Zealand, hot spring geochemistry and microbial composition are strongly influenced by magmatic inputs of heat and volatiles to fluids^13–16^. Recent work from Costa Rica shows that hot spring geochemical and microbial compositional differences reflect geological differences associated with the dip angle of the subducting slab along this volcanically active arc segment, suggesting a connection between tectonics and hot spring microbial ecology^17–19^. Studies that target hot springs more distal to active or recent magmatism are far fewer. For example, work on mesothermal carbonic springs in the western US suggests that crustal and mantle fluid inputs contribute to geochemical conditions that influence spring microbial composition^20^, and the microbial community and geochemical compositions of hot springs associated with fault-controlled geothermal systems in the Tibetan Plateau appear to be governed primarily by spring temperature^21,22^.

**Fig. 1:**
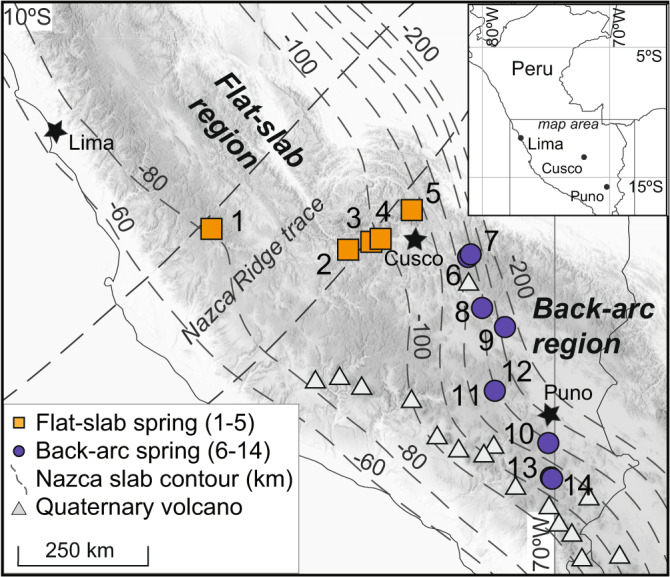
Location of the 14 investigated hot springs in the flat-slab and back-arc regions of Peru. Subducting Nazca slab contours after Hayes et al.^47^. Note the slab steepening southeast of the subducting Nazca ridge trace and the appearance of active arc volcanoes.

Continental arcs, characterized by variations in subducting plate geometry and lithosphere–asthenosphere interactions, host hot springs that provide a natural laboratory to compare their geochemical and microbial composition as a function of the tectonic environment. Today, the western margin of South America is characterized by segments of normal dip subduction and active arc volcanism that are separated by three amagmatic segments with flat-slab subduction^23–25^. During flat-slab subduction the down-going oceanic plate is in contact with the overriding continental lithosphere^23^, and this geometry eliminates the asthenospheric wedge and processes that lead to arc magmatism, resulting in large volcanic gaps along the plate margin. The longest of these flat-slab segments in the Andes underlies most of Peru, located between active arc volcanism in Ecuador and the subducting Nazca ridge along its southern margin. South of the Nazca ridge, the slab steepens to normal subduction with arc volcanism in the western Cordillera and the back-arc of the Altiplano Plateau^26,27^ (Fig. 1). Quaternary volcanism in the back-arc is restricted to small-volume alkaline intrusions and lava flows^28^. The present-day back-arc likely experienced an episode of mid-Cenozoic flat-slab subduction^26^, demonstrating the cycling nature of subduction along continental arcs.

In this contribution, we present new geochemical, temperature, and 16S rRNA gene data from 14 hot springs along an ~800 km, roughly subduction zone parallel transect from the flat-slab to back-arc setting in the Peruvian Andes (Fig. 1). The springs investigated in this study are not directly associated with known magmatic-hydrothermal systems associated with active arc volcanoes. Prior work in this region using hot spring helium and carbon isotope geochemistry has shown that the mantle-to-surface flux of volatiles is controlled by the subduction zone geometry^29,30^. These studies illuminate how cycles of flat-slab subduction influence geofluid migration through the continental lithosphere and provide the foundation for our new work. Here we evaluate how differences in plate boundary geometry and associated tectonic processes influence the diversity and putative function of microorganisms observed at hot springs.

## Results

### Spring geochemistry

**Fig. 2:**
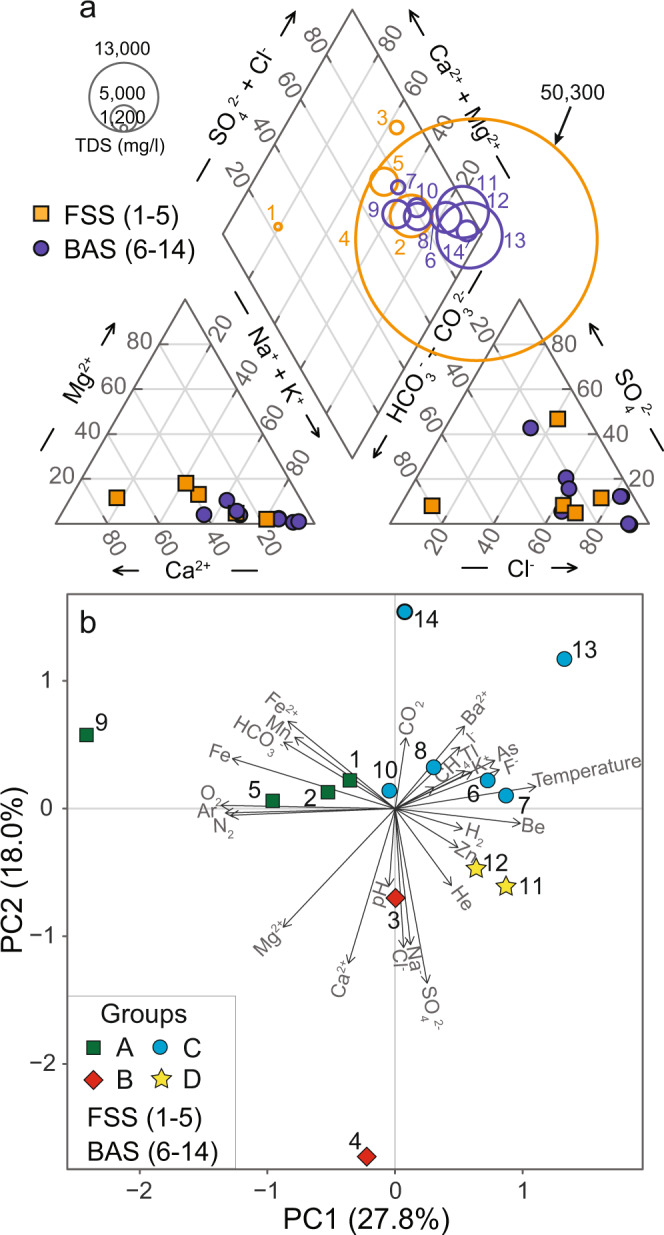
Geochemistry of 14 hot springs in the flat-slab and back-arc regions of Peru. **a** Piper diagram showing the major ion chemistry and TDS for flat-slab springs (FSS) and back-arc springs (BAS; note Sp. 4 TDS). **b** Principal component analysis (PCA) based on the measured aqueous, gas, and isotope geochemistry. Springs separate into four groups FSS into A&B; BAS into C&D, with one major exception (Sp. 9). This lower temperature BAS falls into PCA group A.

Hot spring locations varied from single spring discharges to multiple vents that range in size from <1 to ~10 m^2^. Many springs actively degas (bubble) and all are associated with varying volumes of travertine deposition. The 14 hot springs sampled have a narrow pH range (5.3–6.8) and exhibit a wide range in surface temperature and elemental composition (Supplementary Tables 1–3). With one exception (Sp. 9, 16.6 °C), hot springs in the back-arc region are higher temperatures (43.2–80.5 °C) than those located in the flat-slab region (20.2–45.4 °C). Spring alkalinity as HCO_3_^−^ varies from 68 to 1751 mg L^−1^ with no observed patterns between the regions. Back-arc springs (BAS) are predominantly Na–Cl type waters, whereas flat-slab springs (FSS) are heterogenous ranging from Na–Cl type to Ca–HCO_3_ and Ca–SO_4_ water types (Fig. 2a). BAS is also overall higher in total dissolved solids (2617–12,826 mg L^−1^) than FSS (1344–8193 mg L^−1^), except for one outlier (Sp. 4) located near shallow salt deposits. Dissolved gases are dominated by CO_2_ (21–97 mol%) and N_2_ (2.5–61 mol%), with variable and minor to trace concentrations of O_2_, Ar, He, H_2_, and CH_4_ (Supplementary Table 4). BAS have higher dissolved CO_2_ than flat-slab springs, and CH_4_ is only detected in the back-arc. A PCA using water, gas, and isotope geochemistry identifies four geochemical groups designated A–D (Fig. 2b).

### Spring microbial composition

**Fig. 3:**
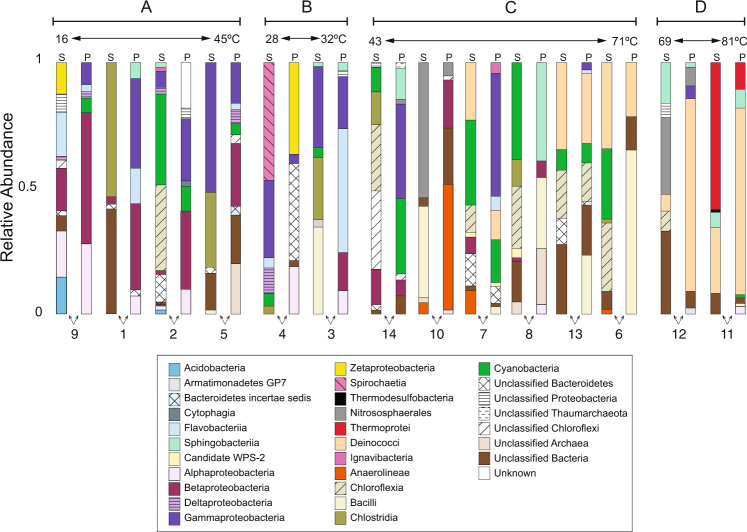
Hot spring relative abundance of 16S rRNA gene operational taxonomic units (OTUs). The sediment (S) and planktonic (P) community of each hot spring is organized by geochemical group (**A**–**D**; as determined using PCA), and by increasing hot spring temperature in each group. Only OTUs that were ≥1% abundance in any one community were considered. Results are presented at the taxonomic class level.

A total of 53,479 unique 16S rRNA gene OTUs are identified in the 14 paired spring planktonic and sediment samples. A total of 210 OTUs are observed with ≥1% relative abundance in any one spring sample (Supplementary Table 5). Of these 210 OTUs, 90% and 10% are affiliated with Bacteria and Archaea, respectively (Supplementary Fig. 1). Out of the 12 bacterial phyla identified, Bacteroidetes, Firmicutes, and Proteobacteria compose 46% of these OTUs. The two identified archaeal phyla are Crenarchaeota and Thaumarchaeota. At the class level, springs at different temperatures host distinct archaeal and bacterial communities, with the planktonic and sediment communities at each spring exhibiting ~10–60% overlap in identified taxa with a wide range in relative abundances (Fig. 3). Proteobacteria comprise over half of the bacterial communities and include the Alpha-, Beta-, Gamma-, Delta-, and Zeta-proteobacteria subclasses. The two observed archaeal classes, Nitrososphaerales and Thermoprotei, are only present in the BAS (Sp. 10, 11, and 12).

## Discussion

### Correlation between microbial composition and hot spring environment

**Fig. 4:**
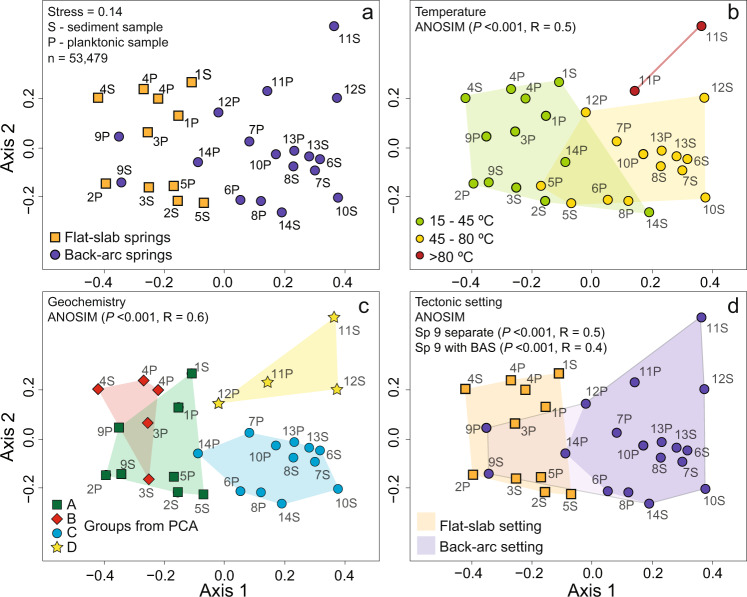
Multivariate statistical analyses of hot spring 16S rRNA gene data. **a** Non-metric multidimensional scaling ordination based on Bray–Curtis dissimilarity for all 16S rRNA gene operational taxonomic units (OTUs, *n* = 53,479) in the 14 springs, with the planktonic and sediment community samples designated. Also the color coding links to the geographic location of the springs shown in Fig. 1. **b**–**d** Analysis of Similarities (ANOSIM) results for treatments overlain on this ordination based on **b** spring temperature range, **d** geochemistry (principal component analysis groups), and **d** tectonic settings. Note that Sp. 9 is an outlier to other BAS, and clusters with FSS on the ordination. The tectonic setting ANOSIM shown in **d** treats Sp. 9 separately as its own group (*P* < 0.001, *R* = 0.5), and as included with the back-arc (*P* < 0.001, *R* = 0.4).

Statistical comparisons of 16S rRNA gene OTU data and environmental variables (geochemistry, temperature, location) are applied to evaluate the controls on Peru hot spring microbial diversity. The NMDS ordination for dissimilarity matrices based on the complete (*n* = 53,479) and the ≥1% relative abundance (*n* = 210) OTU data sets including both planktonic and sediment samples yield acceptable stresses of 0.14 (stress > 0.3 arbitrary)^31^ (Fig. 4a; Supplementary Fig. 2, Table 6). Ordinations considering the planktonic and sediment samples independently, using the full OTU data set, yield stresses of 0.09 and 0.05, respectively (Supplementary Fig. 3, Table 6). Visual inspection of the NMDS ordination plots suggests that the relative distance between samples is not random and that patterns of clustering are correlated to groupings of springs based on surface temperature, water geochemistry, and geological setting. ANOSIM was used to test for statistically significant differences in microbial communities between these categories, here defined by low-range (<45 °C), mid-range (≥45–80 °C), and high-range (≥80 °C) temperature groups, four geochemistry groups (A – D from the PCA, Fig. 2b), and two geographic regions based on the underlying geologic framework (i.e., flat-slab vs. back-arc tectonic setting). A suite of ANOSIM tests using the combined planktonic and sediment communities and considering these independently confirm statistically significant differences in microbial community based on temperature (*R* = 0.3–0.6, *P* < 0.001), geochemistry (*R* = 0.5–0.7, *P* < 0.002–<0.001), and tectonic setting (*R* = 0.4–0.5, *P* < 0.001) (Fig. 4b–d; Supplementary Table 6). Due to the outlier nature of 9 S/P (Fig. 4a), the tectonic tests were conducted two ways: including 9S/P in the back-arc group and as an independent group (Fig. 4d; Supplementary Fig. 4). ANOSIM using geochemical groups based on the major ion chemistry (Fig. 2a) did not yield statistically significant results (Supplementary Table 6). Partial Mantel tests that compare community 16S rRNA gene OTU distance to one of the other categories (e.g., geochemistry), while controlling for the influence of a third (e.g., temperature), corroborate the ANOSIM results and suggest that these parameters significantly influence the composition of microbial communities (Supplementary Table 7). For example, this test shows that the geological setting is still a statistically significant factor for microbial community differences when controlling for spring temperature.

While temperature is significantly correlated to microbial community composition, paired comparison of spring communities from different geologic settings, but with similar temperatures, suggests that other factors contribute to overall community compositional variation. For example, a comparison of the highest temperature FSS samples (2S/P, *T* = 39.9 °C and 5S/P, *T* = 45.4 °C) against those from the BAS setting with similar temperatures (14S/P, *T* = 43.2 °C), reveals considerable variation along NMDS axis 1 (Fig. 4a). Specifically, the 14S community is more similar to BAS communities regardless of temperature, while the 14P community is intermediate to BAS and FSS communities (Fig. 4b), suggesting that temperature did not solely control community variation across geologic setting. Likewise, the lowest temperature samples from the BAS setting (9S/P, *T* = 16.6 °C) were outliers among the BAS communities (Fig. 4), but were not most similar to FSS communities from springs with the most similar temperature (1P/S, *T* = 20.2 °C), but rather to communities from higher temperature FSS springs (i.e., 2S/P and 3S/P).

Similarly, geochemistry is also significantly correlated to microbial community differences (Fig. 4c), but patterns indicate that other factors are at play in these different geologic environments. In contrast to temperature, there is less variation within the individual geochemical groups along NMDS axis 1, and most of the distance is between the FSS (groups A and B) and BAS (groups C and D). The separation between groups C and D is only along axis 2 and controlled by samples 11S/P (*T* = 69 °C) and 12S/P (*T* = 81 °C), which are from different emanations at the same hot spring complex. In this case, the local differences in geochemistry associated with an overall higher-temperature setting may distinguish these from the other BAS locations. Similarly, groups A and B composed of mostly FSS (except for Sp. 9) overlap with some minor separation along axis 2 controlled by 4S/P, collected from an anomalously saline spring (Fig. 4c). As described previously, sample 9S/P is an outlier amongst the BAS, and geochemically falls in group A with other FSS. This spring is anomalous in several ways compared to other BAS. It is the lowest temperature spring in the back-arc, yet has the highest bicarbonate content, with vigorous gas emissions dominated by N_2_ (Supplementary Tables 3, 4). We interpret these examples as secondary, local geochemical controls imparted on divisions dominated by fundamental differences between the back-arc and flat slab settings.

Results also show that hot spring pH is not a primary driver of microbial composition in these springs, and this is consistent with the narrow pH range (6.1 ± 0.4) of the investigated springs. This contrasts with magmatic-hydrothermal systems, where springs exhibit a range of pH (<1.0 to near 10.0) due to the flux of volatiles (e.g., sulfide) into hydrothermal systems, the oxidation of those volatiles to form acid (HSO_4_^-^), and subsequent rock–water interactions^14^. In magmatic-hydrothermal systems, pH is the primary factor influencing microbial diversity and function in hot springs^8,9,12,15,32^.

Our observations in Peru indicate that with few exceptions, the hot spring microbial communities in the flat-slab and back-arc tectonic settings are distinctly and statistically different (Fig. 4d). The results imply that the tectonic differences associated with the geometry of the subduction zone (i.e., flat-slab vs. back-arc configurations) are likely responsible for the spring geochemical and temperature differences that influence the microbial community composition (Fig. 4b, c).

### Controls on hot spring temperature and geochemistry

In regions of mountainous topography, hot spring temperatures are a function of geothermal fluid circulation depth in fracture zones and interaction with shallow and cooler groundwater circulation^2^. Thus, spring water temperatures are not necessarily correlated with subsurface geothermal fluid temperatures or heat flow. To gain further insight into regional patterns of geothermal fluid temperature, we apply geothermal exploration techniques using spring geochemistry to compute subsurface “reservoir temperature estimates” (RTEs). We compare the empirical and theoretical cation (Na–K, Na–K–Ca, Mg-corrected Na–K–Ca, K–Mg) geothermometers^33–35^ with results from multicomponent geothermometry using GeoT^36,37^ (see Supplementary Methods). Processes such as mixing and dilution, degassing and boiling, and partial re-equilibration during fluid ascent to hot springs often lead to unreliable results in the cation thermometers. Multicomponent geothermometry that thermodynamically “reconstructs” the deep geothermal fluid composition can overcome some of these limitations and improve RTEs^36^.

The GeoT simulations yield RTEs from 66 to 105 °C for FSS, which are similar to the K–Mg, Na–K–Ca, or Mg-corrected Na–K–Ca estimates (Supplementary Figs. 6, 7, and Table 8). The Na–K geothermometer, which is most applicable to geothermal fluid temperatures >150 °C, overestimates RTEs for FSS. GeoT RTEs for the BAS range from 116 to 256 °C, consistent in some springs with estimates using the Na–K geothermometer. The results from both methods of geothermometry indicate that lower geothermal reservoir temperatures exist over the flat slab region, whereas higher temperatures exist in the back-arc, and this is generally consistent with hot spring surface temperature patterns.

These RTEs also have implications for the observed spring geochemistry and corroborate the geochemical trends (Fig. 2a) and divisions identified by PCA (Fig. 2b). The dataset suggests a trend of “immature” to “partially equilibrated” waters^34^ with respect to chemical equilibration between circulating geofluids and minerals along subsurface flow paths (Supplementary Fig. 5). Consistent with their restricted Na–Cl type composition and inclusion of elevated levels of redox-sensitive trace elements and gases, the BAS show a higher degree of geothermal fluid–rock equilibration at higher temperatures than the more chemically variable FSS. Springs in the flat-slab region are geochemically immature due to more mixing and dilution with shallow groundwater and incomplete re-equilibration with near-surface, lower-temperature rocks.

Published isotope geochemistry^29,30^ from these hot springs are consistent with the interpreted differences in subsurface temperatures and fluid–rock interactions between flat slab and back-arc regions presented here. Hiett et al.^30^ show that water δ^18^O and δ^2^H values from these springs are derived from the infiltration of meteoric water consistent with hot springs globally^38^, but differences are apparent between the flat slab and back-arc regions. FSS water stable isotopic values suggest derivation from local meteoric water infiltration with minimal high-temperature water–rock isotopic exchange. In contrast, δ^18^O and δ^2^H values for BAS indicate varying degrees of isotopic exchange at elevated temperatures between infiltrating meteoric water and bedrock and/or mixing with deep geofluids^30^. In both regions, the helium isotope (^3^He/^4^He) ratios and the δ^13^C values of dissolved CO_2_ derive from a mixture of mantle and crustal sources^29,30^. BAS geochemistry requires a higher flux of mantle-derived volatiles than the FSS, attributed to the presence of an asthenosphere directly below the continental lithosphere and associated higher heat flow. Mantle-to-surface flux of mantle volatiles is also evident in FSS, but they exhibit relatively more contributions from crustal components.

We also note that the broad geochemical and isotopic differences between the FSS and BAS are not consistent with local patterns in bedrock geology. The bedrock geology along the hot spring sample transect is complex and not distinctly different in these two broad regions and is the product of depositional, magmatic, and deformational processes associated with the protracted development of this convergent plate boundary^39–41^. Collectively, we argue that the major geochemical differences and trends observed between the FSS and BAS systems (e.g., Fig. 2) are governed by large-scale, plate-boundary controls on geothermal fluid temperature related to the geological setting and that the microbial community is adapting to this on the same large scale (Fig. 4). However, local heterogeneities in lithology and groundwater mixing impart another layer of complexity and variability in the spring environments. It is likely that these more localized processes are responsible for the geochemical sub-groupings in the two tectonic settings (e.g., Fig. 2b), and some of the differences in microbial community composition and relative abundances between springs with similar environmental conditions and geologic settings (e.g., Figs. 3 and 4).

### Connections to tectonic setting

**Fig. 5:**
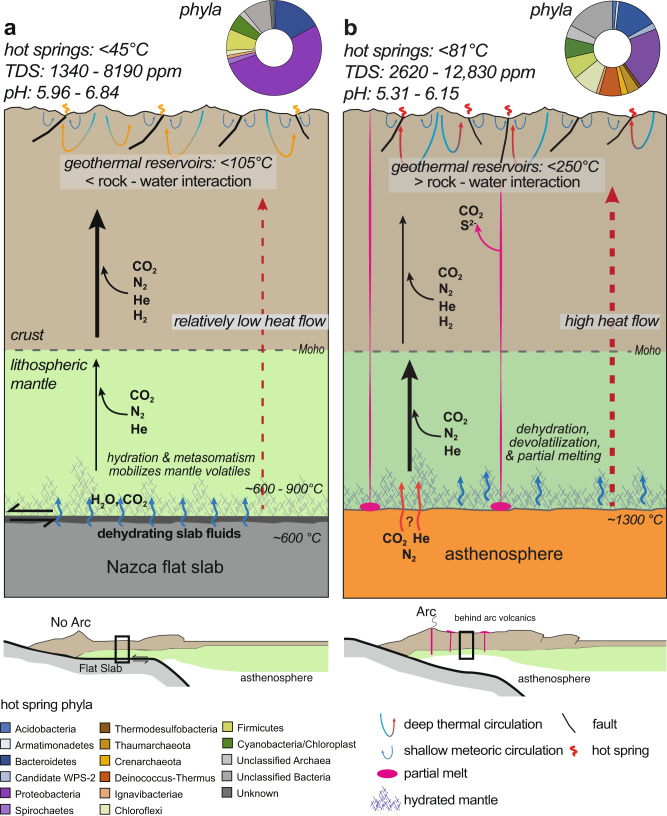
Schematic diagrams of the flat-slab and back-arc tectonic settings highlighting the major geologic controls on the regional hot spring temperature and microbial ecology. The interpretations of volatile flux from the different mantle and crustal reservoirs in the **a** flat-slab and **b** back-arc settings are modified from Hiett et al.^30^. The schematic plate boundary cross-sections for the flat-slab and back-arc configurations are after Hoke and Lamb^48^.

The large-scale tectonic differences between the flat-slab and back-arc region of Peru are attributed to the geometry of the subducting Nazca plate, and the presence or absence of the asthenosphere in direct contact with the overriding continental lithosphere (Fig. 5). The flat-slab segment is underlain by the cool, subducting Nazca slab that is in direct contact with the continental lithosphere. South of the Nazca Ridge where the slab is subducting steeply, hot asthenosphere is present below the South American lithosphere^42^. These differences support distinctly different heat flow patterns. In the flat-slab region, heat flow estimates range from 30 to 50 mW m^−2^ which, in combination with the lack of active magmatism, are consistent with a cool mantle^43^. The back-arc region of the Altiplano Plateau has much higher heat flow values of ~85 mW m^−2 ^^43^ that are attributed to heat flow from the asthenosphere and the efficient transport of this heat into the crust^44^.

The deep-seated controls on heat input into the continental lithosphere along this distinct transition in subduction style have been in place for millions of years supporting geothermal fluid circulation in the upper crust. On these time scales, hot spring emanations are more transient environments that are impacted by dynamic surface processes but are ultimately supported by long-lived geothermal fluid flow. We contend that the differences in plate-boundary processes that influence fluid and heat flow produce the crustal temperature regimes observed in the flat-slab and back-arc settings that influence hot spring environmental conditions on the regional scale (Fig. 5). We demonstrate that the patterns in Peru’s hot spring biodiversity are not random and correlate with the differences in tectonic setting, as the result of microbial diversification to inhabit relatively narrow geochemical and temperature realms (e.g. refs. ^8–10^). We acknowledge that local differences in near-surface conditions (e.g., lithology, groundwater mixing) impart additional variability in the spring environment that will influence the microbial composition, but these appear secondary in Peru hot springs across this geologic transition. Our findings corroborate the recent work from the Costa Rica convergent margin^17,19^ that attributed geochemical and microbiological differences in hot springs to variation in the steepness of the subduction oceanic slab along a volcanically active arc segment. Our results extend these emerging tectonic linkages to the Andean convergent margin and volcanic gaps due to flat slab subduction.

## Conclusions

Our new results from 14 hot springs in the flat-slab and back-arc regions of the Peruvian Andes reveal that spring microbial community composition is distinctly different in these two tectonic settings, and correlates to patterns in temperature and geochemistry. Peru hot springs are slightly acidic to circumneutral with a wide range in temperature in composition. This contrasts with active magmatic-hydrothermal systems, where extremes in hot spring pH and temperature, due to shallow magmatism, influence microbial diversity. In Peru, spring and geothermal system temperature patterns are linked to lithosphere–asthenosphere interactions, specifically the presence of cool oceanic lithosphere (flat-slab setting) versus hot asthenosphere (back-arc setting) directly below the continent. Variations in the subduction angle vary spatially on geological time scales (10s of millions of years) along 1000s of kilometers of convergent margin, and we suggest that plate boundary processes strongly influence the distribution and composition of microbial life in hot spring emanations. To the extent that these processes have played out over millions of years, such observations suggest the potential for the tectonic regime to exert substantial influence on the diversification of microbial life in these environments.

## Methods

Fourteen hot spring systems were investigated along a roughly NW–SE transect of the Peruvian Andes that traverses the present-day flat-slab subduction segment into the back-arc of the active volcanic arc in southern Peru (Fig. 1). Hot springs (Sp.) 1–5 are located in the flat-slab region (flat-slab springs, FSS) and hot springs Sp. 6–14 are located in the back-arc (back-arc springs, BAS). Water and gas samples were collected at the spring source for major and trace element and dissolved gas analyses. For locations with more than one spring vent, the location with the highest temperature and total dissolved solids (estimated from the specific conductance) was sampled. In the field, specific conductance, pH, and temperature were measured with a portable meter, and Fe(II) and S^2−^ concentrations were determined using a portable spectrophotometer and reagents. Samples of filtered (0.2 µm) spring water and sediments were collected from each location for molecular analyses. Genomic DNA extraction, polymerase chain reaction (PCR) amplification of 16S rRNA genes, sequencing, and bioinformatics analyses followed methods in Colman et al.^45^. 16S rRNA gene operational taxonomic units (OTUs) were assigned at a sequence similarity of ≥97%. The Supplementary Methods provide comprehensive details on the field sampling and analytical techniques.

Multivariate statistical analyses were used to compare environmental variables, tectonic settings, and the composition of 16S rRNA gene OTUs. All statistical analyses were conducted in RStudio (version 1.1.463) using several packages for data visualization and analysis, and these are described comprehensively in the Supplementary Methods. A principal component analysis (PCA) of temperature, pH, dissolved gases, and water geochemical (major and trace element) composition data was created to address linear relationships between samples and geochemical variables. Non-metric multidimensional scaling ordination (NMDS) used a Bray–Curtis dissimilarity matrix for combined sediment and planktonic 16S rRNA gene OTU data. The NMDS ordination provides a visualization tool to assess the similarity between sample microbial composition based on the proximity (i.e., closer are more similar) between samples on the ordination. The Analysis of Similarity (ANOSIM) test was used to determine the statistical significance of 16S rRNA gene OTU similarity as shown on the NMDS ordination to environmental and geological setting groupings. In addition, the partial Mantel test, which determines the correlation between two matrices while controlling for the influences of a third, was applied to assess correlations between the community 16S rRNA gene OTU data and environmental and geographic variables. The test was used to evaluate the correlations between community 16S rRNA gene OTU dissimilarity matrix and (1) temperature while controlling for tectonic setting, (2) geochemistry, while controlling for geographic location, (3) tectonic setting while controlling for temperature, and (4) geochemistry, while controlling for temperature.

### Reporting summary

Further information on research design is available in the Nature Portfolio Reporting Summary linked to this article.

### Supplementary information

Supplementary Information

Reporting Summary

## Data Availability

Data generated and analyzed in this study are publically available in the Figshare repository: 10.6084/m9.figshare.22233862.v1^46^.
