# Supplementary material for: Tectonic settings influence the geochemical and microbial diversity of Peru hot springs
Source: Commun Earth Environ. 2023 Apr 11;4(1):112. doi: 10.1038/s43247-023-00787-5 (PMC11041657; doi:10.1038/s43247-023-00787-5)
Supplement: Supplementary file 1 — Supplementary Information [file 43247_2023_787_MOESM1_ESM.pdf]

Supplementary Information: Tectonic settings influence the geochemical and microbial diversity of Peru hot springs

HE Upin[1], DL Newell[1,*], DR Colman[2], and ES Boyd[2]

[1] Dept. of Geosciences, Utah State University, Logan, UT, USA

[2] Dept. of Microbiology and Cell Biology, Montana State University, Bozeman, MT, USA

* Corresponding author

Supplementary Methods

Spring locations: This study investigates fourteen hot spring systems located along a roughly NW-SE transect of the Peruvian Andes (Fig. 1, main text, generated using ArcMap 10). These springs were selected based on existing geochemical data sets, geographic location, accessibility, and tectonic setting [1,2]. The 14 springs are located in the Peruvian provinces of Ayacucho, Apurimac, Cusco, Puno, and Moquegua. Our aim was not to simply target extreme conditions, but rather to capture a range of aqueous and gas chemistries, temperatures, and geological settings. Many spring locations are actually a cluster of individual emanations. In those cases, sampling was based on temperature and specific conductance measurements so that the spring with the least amount of near-surface mixing with dilute and low-temperature groundwater could be targeted as assessed in the field by measurements of the highest temperature and highest specific conductance.

Water and gas sampling: Spring sampling and field measurements were conducted as close to the spring source as possible while maintaining safe distance from hot fluids and unstable ground. Water and gas samples from large high temperature springs were collected using a funnel and tubing attached to a 2.5 m pole. Water samples for cations were field-filtered into 60 mL wide-mouth Nalgene® HPDE bottles using 0.45 µm syringe filters (Thermo Scientific, surfactant-free cellulose acetate). Filtered cation samples were later acidified using 0.5 mL concentrated trace-metal grade nitric acid. Samples for anions and alkalinity determinations were collected unfiltered into 125 mL wide-mouth Nalgene® bottles with no head space. In cases where waters were particularly turbid, these samples were also filtered. Gasses from bubbling springs were collected into 12 in. by 3/8 in. ID copper tubes via polyethylene tubing and an inverted funnel, and cold sealed using metal clamps following methods adopted from Hilton et al. [3]. The funnel and tubing were flushed with spring water and then gasses before sampling to prevent atmospheric contamination. Samples were kept cool and dark prior to analyses.

Microbiology sample collection: Water (planktonic cells) and sediments (sediment-associated cells) from springs were collected for use in DNA extraction and sequencing. For planktonic cells, 1–3 L of water were passed through 0.2 µm Sterivex® filters using a 140 mL syringe that was flushed with spring water prior to use. After filtration, a volume of air was passed through the Sterivex® filter to remove any remaining water from the filter, which was then stored in a sterile

Whirl Pak® bag. Microbial mat and sediment samples were collected using a flame sterilized metal spoon and placed into 15 mL centrifuge tubes. All microbiology samples were collected in either duplicate or triplicate and stored at room temperature for 5–15 days until shipment back to Utah State University (USU). Once back in the lab, samples were frozen to -80 ºC.

Field Measurements: Specific conductance, pH, and temperature measurements were collected in the field using an Orion® portable meter. Field spectrophotometer measurements of ferrous iron and sulfide were adapted from the HACH 8146 1,10-Phenanthroline Method and the HACH 8131 USEPA Methylene Blue Method, respectively [4]. Using these methods, the dynamic range for ferrous iron (Fe^{2+}) is 0.02 to 3.00 mg/L and 5 to 800 μg/L for sulfide (S^{2-}). Samples were collected as close to the source as possible, and readings were taken immediately after collection. Samples were initially analyzed undiluted. If concentrations exceeded the maximum range for the method, the samples were diluted as necessary to within the detectable range. Field dilutions were accomplished using bottled water and a 50 ± 0.5 mL graduated cylinder.

Water and gas geochemical analyses: Dissolved gas composition was measured in 10 of the 14 springs using gas chromatography quadrupole mass spectrometry (MS) at the University of New Mexico Fluid and Volatiles Lab. Gas composition was reported as a mole percent and converted to dissolved molar concentrations using published Henry's Law constants [5]. Major ions and trace elements were measured in 14 springs by inductively coupled plasma MS (ICP-MS) and ion chromatography (IC), respectively, at the USU Water Research Lab. Cation and anion samples were volumetrically diluted, as needed, for measurement on the ICP-MS and IC. Alkalinity was measured as total carbonate alkalinity in the USU Geochemistry Lab using colorimetric titration [6].

DNA extraction and 16S rRNA gene sequencing: Methods for genomic DNA extraction, polymerase chain reaction (PCR) amplification of 16S rRNA genes, sequencing, and informatics were conducted according to the methods outlined in Colman et al. [7]. Briefly, filters were cut open using a sterilized jewelry saw, removed from their housing, and peeled from the filter cartridges using sterilized tweezers. Filters or ~0.35 g of sediment were placed into FastDNA SPIN kit bead-beating tubes (MP Biomedicals) with sterilized tweezers or spatulas, respectively. DNA was extracted from each using the FastDNA SPIN Kit for Soil using previously described methods in Boyd et al. [8] and yielded a total of 29 microbiology samples (one filter was split into two tubes due to a large amount of visible biomass). Genomic DNA was quantified using a Qubit fluorescence assay and fluorometer and roughly 200 ng of DNA were subject to PCR using universal primers (515F and 806R) as previously described in Colman et al. [7]. 16S rRNA gene amplicons were sent to the Molecular Research DNA Lab in Shallowater, Texas, for sequencing utilizing the paired end (2x300 bp) Illumina MiSeq platform.

16S rRNA genes were subjected to analysis using the software program Mothur [9] as previously described by Colman et al. [7]. In short, sequences were filtered, aligned, and trimmed using defined start and end sites based on 85% inclusion of total sequences. Redundant sequences and sequencing errors were detected and removed. Chimeras were identified and removed using

UCHIME [10]. Operational taxonomic units (OTUs) were assigned at a sequence similarity of ≥ 97% using the nearest-neighbor method. The sequence dataset was randomly subsampled to generate 45,226 gene sequences per sample. High abundance OTUs (≥ 1% relative abundance) were subjected to BLASTn analysis to identify the most closely related taxon with a characterized physiology. Adopting this information, a prediction of the metabolism for 16S rRNA gene phylotypes was made for each high abundance OTU.

Statistical analyses: Statistical analyses of geochemical and 16S rRNA gene OTU data were conducted in RStudio (version 1.1.463). A principal component analysis (PCA) of temperature, pH, dissolved gas, and water geochemical composition data was created to address linear relationships between samples and geochemical variables. The PCA was made with the prcomp function in the stats package and graphically displayed with the ggplot2 package.

Multivariate analyses, including non-metric multidimensional scaling ordination (NMDS), were conducted using the vegan package version 2.5-4 [11,12]. The NMDS ordinations were made with the metaMDS function and a calculated Bray-Curtis dissimilarity matrix with three dimensions for combined sediment and planktonic 16S rRNA gene OTU data. We used the envifit function within metaMDS to assess the correlation between environmental parameters and the 16S rRNA gene OTU data community distance matrix. NMDS ordination provides a visualization of the dissimilarity of assemblages (communities) of 16S rRNA gene OTUs between sites. The function ANOSIM, a program in the vegan package, was used to determine the statistical significance of geochemical, temperature, and geographic spring groupings. ANOSIM provides a way to test whether different treatments are statistically significant to community 16S rRNA gene OTU similarity, and returns a P value (considered significant if <0.01 at 99 % confidence) and an ANOSIM R statistic (a metric, in its definition, specific to this program). The R statistic can vary between -1 and 1 and is based on the dissimilarities between and within groups. If the dissimilarity between groups is high but within groups is low, the R statistic will approach 1, indicating the communities within treatment groups are similar but different from one another. An R statistic of 0 implies random grouping and a negative R statistic indicates that the dissimilarity within groups is greater than between groups.

Another way to statistically test for correlations between the community 16S rRNA gene OTU data and environmental variables is the partial Mantel test. This test determines the correlation between two matrices, while controlling for the influences of a third matrix. The function partial.mantel, in the R vegan package, was used to 1) estimate the correlation between community 16S rRNA gene OTU (dissimilarity matrix) and temperature, while controlling for geographic location (tectonic setting in this study), 2) estimate the correlation between community 16S rRNA gene OTU and geochemistry (from the PCA), while controlling for geographic location, 3) estimate the correlation between community 16S rRNA gene OTU and geographic location, while controlling for temperature, and 4) estimate the correlation between community 16S rRNA gene OTU and geochemistry, while controlling for temperature (Supplemental Table 7). This analysis uses the same matrices and groupings from the NMDS ordination and ANOSIM tests.

The function calculates a Mantel r statistic, which is the Pearson's product-moment correlation coefficient, and a *P*-value to evaluate significance. The r statistic falls between -1 and 1, where -1 indicates a negative correlation, 1 indicates a positive correlation, and 0 indicates no correlation.

Geochemical modeling and Geothermometry: Ion balances were calculated with the major ion and trace element chemistry for the 14 springs. The total concentration (in equivalents/L) of cations (c) and anions (a) were used to calculate a percent imbalance using the equation: ([c]-[a])/([c]+[a]) * 100. Geochemists Workbench version 12.0.4 [13] was used to generate a Piper diagram with the measured major ion chemistry [14], and calculate saturation indices for spring components.

Two methods are used to estimate the temperatures of geothermal fluids in the subsurface below each hot spring: conventional cation geothermometry and multicomponent geothermometry. These methods provide "reservoir temperature estimates" (RTEs) that are commonly used in geothermal energy exploration. Here we expand their utility to provide constraints on thermal fluid circulation depth in the different tectono-magmatic regions. Additionally, these RTEs allow comparisons between observed patterns in hot spring temperature, geochemistry, microbial community composition and what we know about regional heat flow in the flat-slab and back-arc regions of Peru.

We report RTEs from the Na-K, Na-K-Ca, Mg-corrected Na-K-Ca, and K-Mg geothermometers [15-18] computed using the Powell and Cumming [19] spreadsheet tool. Dissolved silica measurements are not available for these hot springs, so the suite of silica geothermometers could not be used. These geothermometers and "geoindicators" are based on both theoretical thermodynamic constraints and empirical relationships from geothermal fluids with known reservoir temperatures. A primary assumption is that the fluid chemistry derives from chemical equilibrium with minerals present at depth in the geothermal reservoir such as feldspars and other aluminosilicates, and that other processes during fluid ascent have not significantly altered cation geochemistry. The Na-K geothermometer is most applicable to neutral Na-Cl waters with reservoir temperatures much greater than 100 °C and low Ca contents [15,17]. The Na-K-Ca geothermometer may be more appropriate for higher concentrations of Ca [17], but high CO_2 fluids with associated calcite precipitation can be problematic and can lead to temperature overestimates [15]. Similarly, use of the Na-K-Ca geothermometer on Mg-rich waters can overestimate temperatures, and a Mg correction can improve estimates [18]. For a fully equilibrated, "mature" geothermal fluid, the Na-K and K-Mg thermometers should yield the same temperature; however, the K-Mg geothermometer re-equilibrates more rapidly than the Na-K system during cooling and fluid flow to the surface [15]. Therefore, in hot springs the K-Mg geothermometer often tracks incomplete retrograde fluid-rock interaction and mixing with Mg-rich shallow groundwater, and the slow to re-equilibrate Na-K geothermometer may be more representative of the deeper hotter geothermal fluid.

We also report RTEs using the "multicomponent geothermometry" method [20] implemented by the GeoT program [21]. Factors such as mixing and dilution, degassing and boiling, and partial re-equilibration during fluid ascent violate many of the underlying assumptions for the conventional cation thermometers described above, and can lead to disparate and unreliable

results, especially at hot springs. Multicomponent geothermometry thermodynamically "reconstructs" the deep geothermal fluid composition based on mineral equilibrium and account for these physical and chemical modifications, and can improve the reliability of the RTEs [20]. These GeoT estimates are meant to be used in tandem with the conventional cation geothermometers to evaluate subsurface fluid temperature potential.

We calculated reconstructed geothermal fluid temperatures at each hot spring using iGeoT v1.0 that integrates GeoT into iTOUGH2 [22]. Integration with iTOUGH2 allows for automatic parameter estimation of uncertain or unknown input parameters for GeoT. Input parameters that were automatically estimated by iGeoT in these calculations include dissolved Al, dissolved Si, concentration/dilution factors, and CO_2 partial pressure. Aluminum was estimated because it was below minimum detection limits in all samples due to dilution prior to ICP-MS analysis. The detection limit for Al in each sample was used as a starting input concentration that was then optimized by iGeoT. Silica was not included in the analytical suite, and small starting input concentrations (5-15 mg/l) were used in a suite of simulations that automatically estimated dissolved Si. Gas chemistry was measured for all but one hot spring, and CO_2 is the primary gas species that impacts the dissolved ion chemistry. To account for gas (CO_2) loss from the geothermal fluid, the CO_2 partial pressure was optimized, starting with the measured composition. iGeoT also allows specific minerals to be considered in the calculations, and these are primary and alternation phases expected in the geothermal system. Because dissolved silica measurements are not available for the hot springs analyzed herein, two separate simulations were conducted for each spring: one omitting Si and silicate minerals, and one that estimates the Si. Simulations that did not include Si estimated temperatures using fluid equilibration with carbonates and oxides. Simulations that estimated the Si content considered silicates, carbonates, and oxides. The simulations that include Si are most appropriately compared to the cation geothermometer because these are based on fluid-mineral equilibration with silicates.

Results from the cation geothermometry and multicomponent geothermometry calculations are reported in Supplemental Table 8. Comparison of the Na-K and K-Mg systems using the Na-K-Mg "geoindicator" [15] shows that all hot springs fall in the partial equilibration or immature water fields, and thus the Na-K and K-Mg temperatures are not the same (Supplemental Fig. 3). Overall, the back arc springs (BAS) are more "mature" than flat slab springs (FSS) based on this diagram. The Na-K geothermometer should be used with caution but may track the higher temperature history of the most mature waters, exclusively found in BAS. The Na-K system likely yields unreasonably high estimates for immature FSS. As discussed above, the K-Mg geothermometer will reflect retrograde reactions and/or mixing and may underestimate the subsurface fluid temperatures. With a few exceptions, the Na-Ca-K and Mg-corrected Na-K-Ca geothermometers yield RTEs intermediate between the K-Mg and Na-K estimates. There are four springs with K-Mg estimates that are higher than the Mg-corrected Na-K-Ca estimate.

GeoT estimates for the FSS range from 66 to 105 °C or 90 to 110 °C, for simulations that estimate Si and those that do not include Si, respectively. BAS estimates range from 98 to 256 °C

or 116 to 262 °C for simulations that estimate Si and those that do not include Si, respectively. The simulations with and without estimating dissolved Si yield similar RTEs for most springs (Supplemental Fig. 4 & 5). Thus, for the most robust comparison to the conventional cation thermometers (that are based on silicate mineral-fluid equilibration) we use the RTEs based on estimating Si (Supplemental Table 8).

As presented in the main text of this paper, the GeoT RTEs for FSS are most similar to the estimates from the Na-Ca-K, Mg-corrected Na-K-Ca, and K-Mg geothermometers. These fluids have Ca concentrations that are too high, and reservoir temperatures that are too low for appropriate application of the Na-K geothermometer. At 5 of the BAS (Sp. 6-8, 11, 12), the GeoT estimates are similar to modestly higher than the Na-K geothermometer, suggesting that these hot springs discharge from higher temperature geothermal reservoirs. Four BAS (Sp. 9,10, 13, and 14) have GeoT estimates lower than the Na-K geothermometer and more similar to the other cation systems (Supplemental Table 8). Collectively, we consider the RTEs calculate using multicomponent geothermometry as the most reliable for the suite of hot springs presented in this manuscript.

Supplementary References

1 Steinmuller, K. & Huaccan, A. H. Aguas termales y minerales en el centro del Peru, Boletin No 21. 74 (Instituto Geologico Minero Y Metalurgico, Lima, Peru, 1999).

2 Huaccan, A. H. Aguas termales y minerales en el suroriente del Peru, Boletin No 24. 120 (Instituto Geologico Minero Y Metalurgico, Lima, Peru, 2001).

3 Hilton, D., Fischer, T. P. & Marty, B. in *Reviews in Mineralogy & Geochemistry - Noble Gases in Geochemistry and Cosmochemistry* Vol. 47 (eds D. Porcelli, C.J. Ballentine, & R. Weiler) 319-370 (Mineralogical Society of America, 2002).

4 HACH. *HACH Water Analysis Handbook, www.hach.com/wah*, <www.hach.com/wah> (2020).

5 Sander, R. Compilation of Henry's law constants (version 4.0) for water as solvent. *Atmospheric Chemistry and Physics* **15**, 4399-4981 (2015).

6 USGS. *National field manual for the collection of water-quality data*, <http://pubs.water.usgs.gov/twri9A> (2006).

7 Colman, D. R. *et al.* Ecological differentiation in planktonic and sediment-associated chemotrophic microbial populations in Yellowstone hot springs. *FEMS microbiology ecology* **92** (2016).

8 Boyd, E. S. *et al.* Isolation, characterization, and ecology of sulfur-respiring Crenarchaea inhabiting acid-sulfate-chloride-containing geothermal springs in Yellowstone National Park. *Applied and environmental microbiology* **73**, 6669-6677 (2007).

9 Schloss, P. D. *et al.* Introducing mothur: open-source, platform-independent, community-supported software for describing and comparing microbial communities. *Applied and environmental microbiology* **75**, 7537-7541 (2009).

10 Edgar, R. C., Haas, B. J., Clemente, J. C., Quince, C. & Knight, R. UCHIME improves sensitivity and speed of chimera detection. *Bioinformatics* **27**, 2194-2200 (2011).

11 Oksanen, J. *et al.* Vegan: community ecology package. R package vegan, vers. 2.2-1. *R package version 2. 2* **1** (2015).
12 Oksanen, J. *et al.* (2019).
13 Bethke, C. M. The Geochemist's Workbench Release 6.0, Hydrogeology Program, Dept. of Geology, University of Illinois. (2006).
14 Piper, A. M. A graphical procedure in the geochemical interpretation of water analyses. *Transactions (American Geophysical Union)* **25**, 914-923 (1944).
15 Giggenbach, W. F. Geothermal solute equilibria. Derivation of Na-K-Mg-Ca geoindicators. *Geochimica et Cosmochimica Acta* **52**, 2749-2765 (1988).
16 Fournier, R. Chemical geothermometers and mixing models for geothermal systems. *Geothermics* **5**, 41-50 (1977).
17 Fournier, R. O. & Truesdell, A. H. An empirical Na-K-Ca chemical geothermometer for natural waters. *Geochimica et Cosmochimica Acta* **37**, 1255-1275 (1973).
18 Fournier, R. O. & Potter II, R. Magnesium correction to the Na-K-Ca chemical geothermometer. *Geochimica et Cosmochimica Acta* **43**, 1543-1550 (1979).
19 Powell, T. & Cumming, W. in *Proceedings, 35th Workshop on Geothermal Reservoir Engineering.* 1-10.
20 Reed, M. & Spycher, N. Calculation of pH and mineral equilibria in hydrothermal waters with application to geothermometry and studies of boiling and dilution. *Geochimica et Cosmochimica Acta* **48**, 1479-1492 (1984).
21 Spycher, N., Peiffer, L., Finsterle, S. & Sonnenthal, E. GeoT User's Guide, A Computer Program for Multicomponent Geothermometry and Geochemical Speciation, Version 2.1. 42 (2016).
22 Spycher, N. & Finsterle, S. iGeoT v1. 0: Automatic Parameter Estimation for Multicomponent Geothermometry, User's Guide. 56 (Lawrence Berkeley National Lab.(LBNL), Berkeley, CA (United States), 2016).

Supplementary Figures

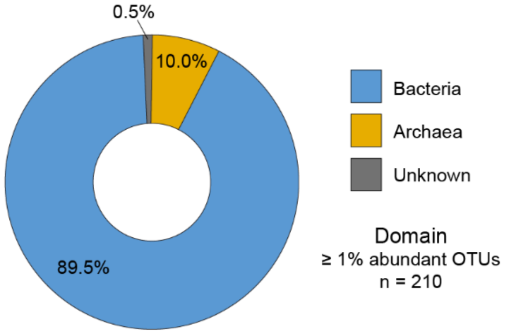

Supplementary Figure 1. Donut diagram showing the distribution of 16S rRNA genes affiliated with Bacteria and Archaea in all 14 springs (both planktonic and sediment community) for the ≥1 % abundance operational taxonomic units (OTUs).

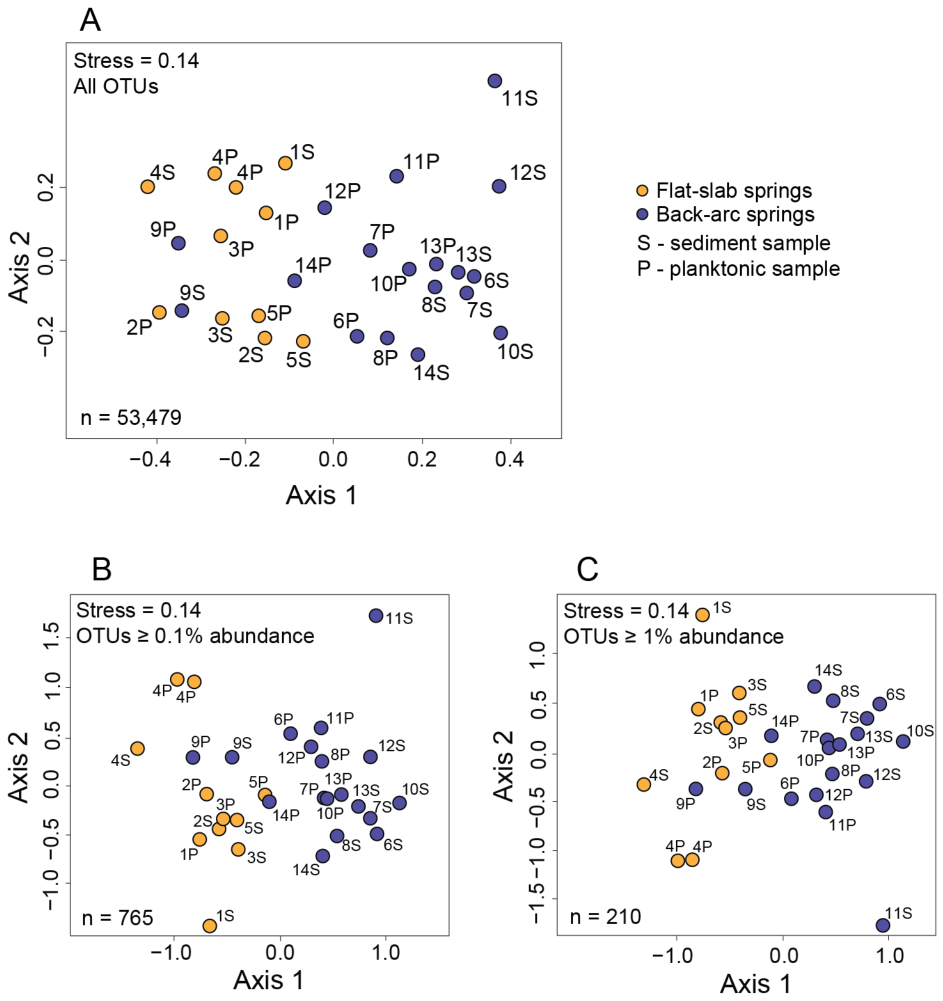

Supplementary Figure 2. Non-metric multidimensional scaling (NMDS) ordination based on Bray-Curtis dissimilarity for (A) all 16S rRNA gene OTUs (n=53,479), (B) the ≥0.1 % OTUs (n=765), and (C) ≥1 % OTUs (n-210) in the 14 springs.

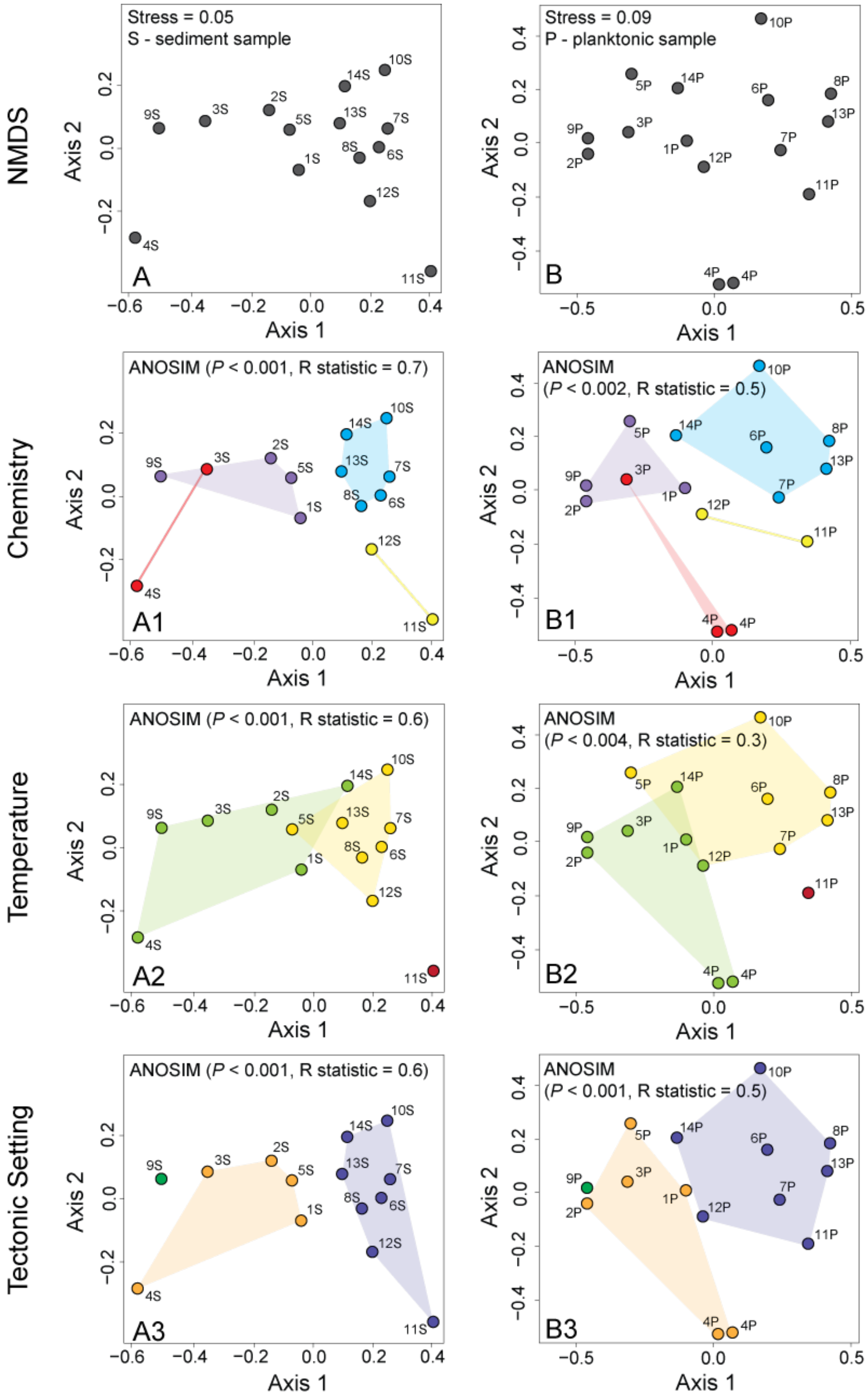

Supplementary Figure 3. Non-metric multidimensional scaling (NMDS) ordination based on Bray-Curtis dissimilarity for all 16S rRNA gene OTUs (n=53,479) for the (A) sediment (S) and (B) planktonic (P) samples. Analysis of Similarities (ANOSIM) results for treatments overlain on these ordinations based on (A1, B1) geochemistry based on Principal Component Analysis groups, (A2, B2) spring temperature range, and (A3, B3) tectonic settings (with 9S and 9P as their own groups).

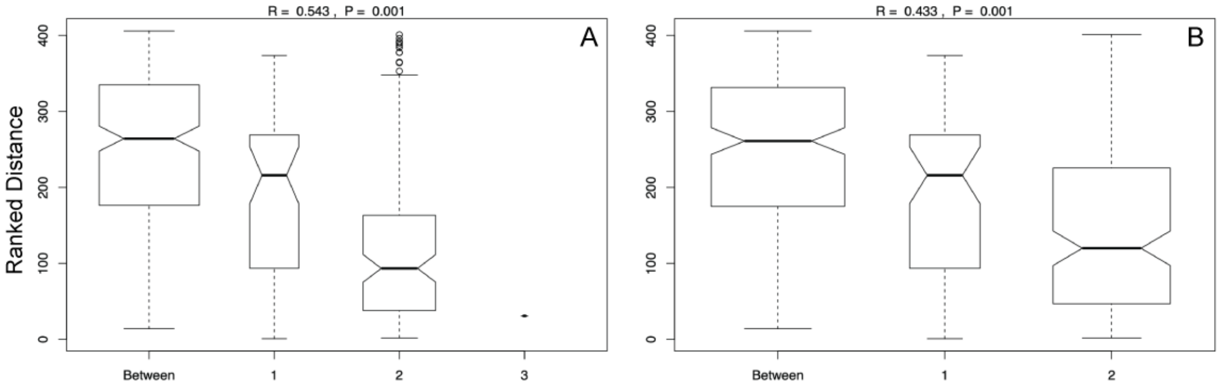

Supplementary Figure 4. ANOSIM boxplots for tectonic setting tests (A) excluding 9S/P from the back arc group, and (B) including 9S/P with the back arc group. 1 = flat slab setting, 2 = back arc setting, 3 = 9S/P group.

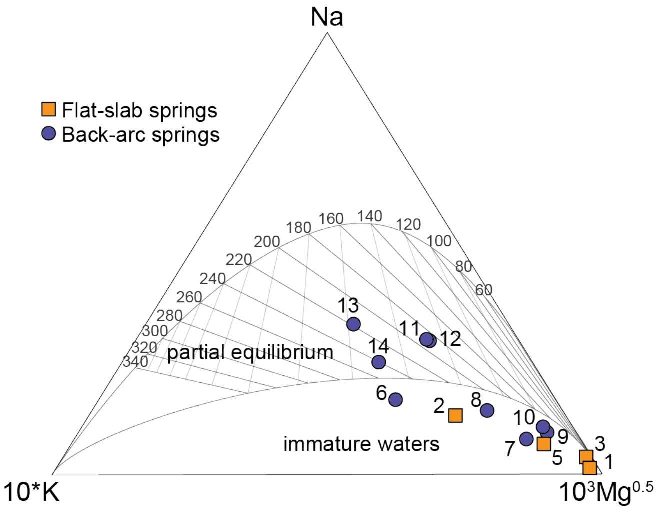

Supplementary Figure 5. Na-K-Mg ternary diagram that compares temperature estimates using the Na-K and K-Mg geothermometers (Giggenbach, 1988). Springs that fall within the "partial equilibration" field provide the most reliable estimates, although retrograde reactions and mixing during fluid ascent and cooling lead to disparities between the two thermometers. The flat slab spring plot closer to the Mg vertex and are geochemically "immature" with respect to these geothermometers.

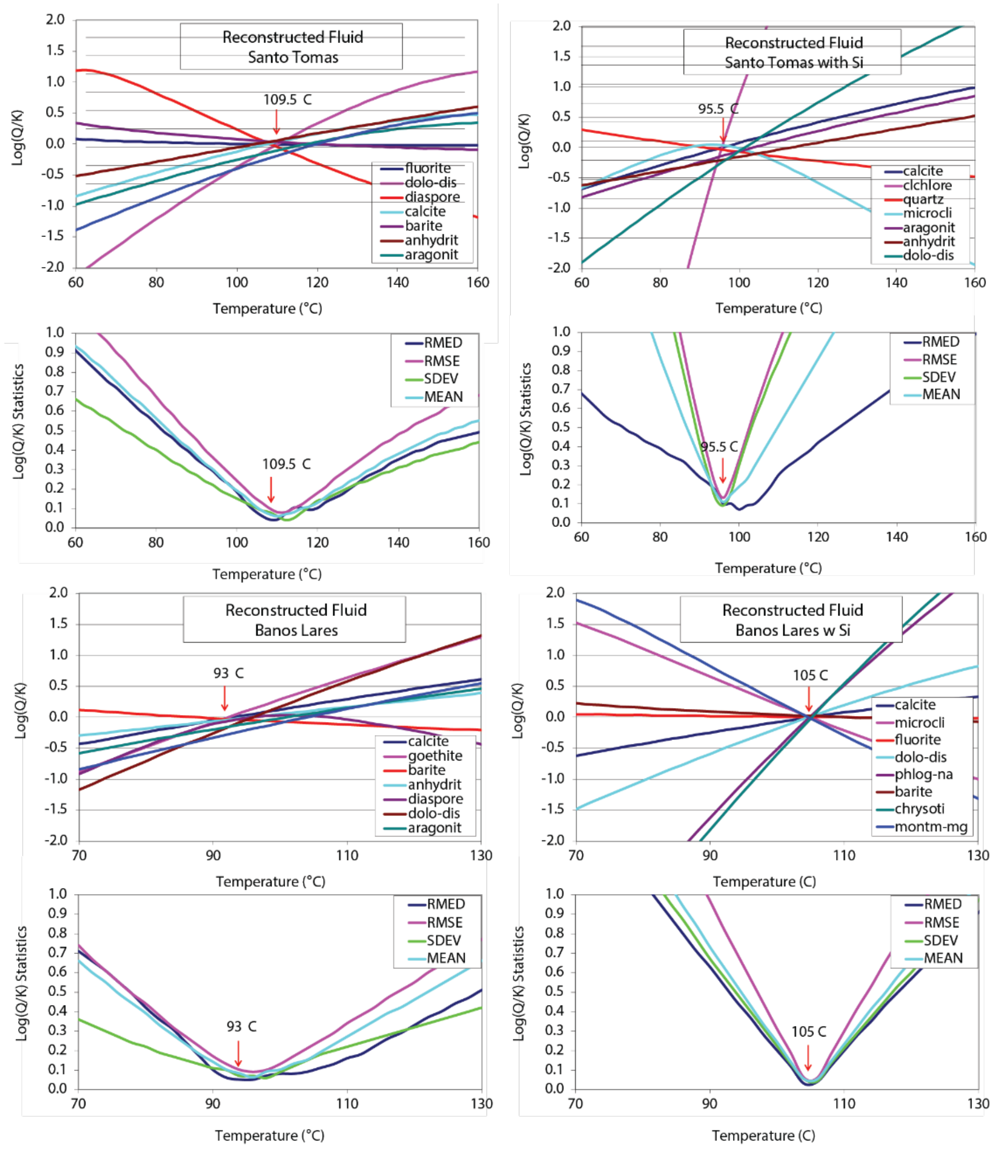

Supplementary Figure 6. Representative GeoT output plots from two hot springs in the flat-slab region for simulations without (left panels) and with Si estimation (right panels). Reservoir temperatures are based on clustering of mineral saturation indices (log Q/K) at 0 (equilibrium), where statistical parameters are at a minimum [21,22].

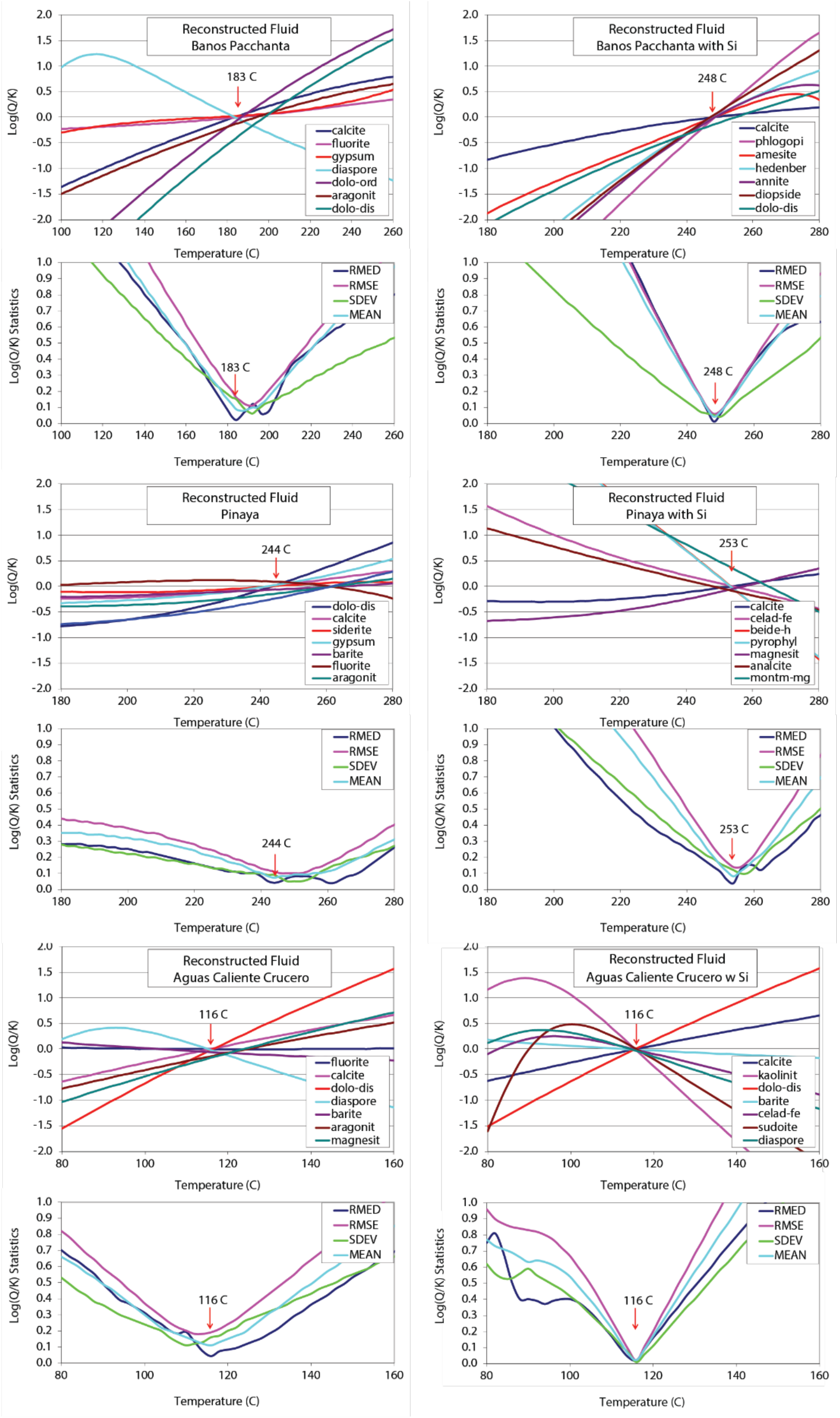

Supplementary Figure 7. Representative GeoT output plots from three 3 hot springs in the back-arc region for simulations without (left panels) and with Si estimation (right panels). Reservoir temperatures are based on clustering of mineral saturation indices (log Q/K) at 0 (equilibrium), where statistical parameters are at a minimum [21,22].

Supplementary Tables

Supplementary Table 1. Spring number, name, geographic location, and measured field parameters

Sp.	Name	Elevation (m asl)	Longitude	Latitude	T (ºC)	pH	Cond. (mS/cm)
**1**	Licapa	4103	-74.87776	-13.36887	20.2	6.53	1.31
**2**	Santo Tomás	1693	-72.94484	-13.65254	39.9	6.12	11.31
**3**	Ccónocc	1850	-72.63866	-13.54313	31.8	6.84	3.26
**4**	Agua termal Sauceda	2308	-72.49839	-13.50098	27.8	6.16	85.80
**5**	Baños termales Lares	3281	-72.05453	-13.11067	45.4	5.96	7.17
**6**	Baños de Upis	4418	-71.27520	-13.75014	71.0	6.54	9.01
**7**	Baños Pacchanta	4308	-71.24201	-13.71736	54.2	5.96	3.27
**8**	Aguas Calientes La Raya	4054	-71.07299	-14.45071	56.4	6.09	7.02
**9**	Pichacani-Santa Rosa	3931	-70.75443	-14.70899	16.6	6.15	6.76
**10**	Baños Termales Collpa Apacheta	4106	-70.14219	-16.26762	54.0	5.98	4.82
**11**	Aguas Calientes-Pinaya	4386	-70.88657	-15.56674	80.5	6.15	15.16
**12**	Aguas Calientes-Pinaya	4379	-70.88768	-15.56689	68.5	6.09	14.61
**13**	Aguas termales Crucero	4572	-70.11648	-16.74175	62.0	5.98	18.76
**14**	Punta Perdida-Pastogrande	4614	-70.08931	-16.76263	43.2	5.31	6.29

Supplementary Table 2. Major ion concentrations (mg/l) for Peru flat-slab and back-arc hot springs.

**Sp.** MRL	**Ca^{2+}** 0.50	**Mg^{2+}** 0.05	**Na^{+}** 0.40	**K^{+}** 0.13	**Cl^{-}** 0.1	**SO_4^{2-}** 0.25	**NO_3^{-}** 0.05	**HCO_3^{-}** 0.05	**F^{-}** 0.05	**Ion Balance (%)**
**1**	225	24.5	70.0	8.15	89.7	77.6	< 0.10	848	1.49	-5
**2**	674	96.3	1923	296	3096	354	0.98	1751	2.26	4
**3**	295	88.0	381	9.95	614	912	0.24	223	1.70	-2
**4**	1649	182	16284	< 130	30023	1954	< 3.5	68	0.00	-5
**5**	585	138	894	100	1915	367	< 0.10	1330	2.10	-1
**6**	266	23.7	1498	263	2671	563	< 0.20	594	4.76	-5
**7**	263	19.5	422	54.0	490	832	< 0.10	532	3.93	-7
**8**	396	42.1	1256	123	1782	623	< 0.10	1015	3.56	1
**9**	434	115	1158	69.5	1938	247	< 0.20	1454	2.57	0
**10**	269	43.0	845	44.5	1159	561	< 0.10	643	2.13	0
**11**	365	33.5	3350	183	5034	900	< 0.50	247	8.34	2
**12**	357	33.4	3240	178	5014	975	3.50	229	8.76	0
**13**	153	26.4	4616	386	6931	70.4	< 0.50	637	6.34	3
**14**	73.1	5.73	1297	144	2174	19.5	< 0.20	198	3.03	0

MRL – minimum reporting limit in mg/l

Supplementary Table 3. Trace element concentration (in ppb) for Peru flat-slab and back-arc hot springs

**Sp.**	I^-	Fe^{2+} *	S^{2-} *	**Fe**	**As**	**Mn**	**Ba**	**Be**	**Ni**	**Zn**	**Cr**	**Co**	**Cu**	**Se**	**Sb**	**Tl**	**Pb**
MRL				5.00	0.25	0.50	1.25	0.06	1.00	2.00	0.10	0.16	0.63	0.13	3.00	0.10	0.25
1	-	0.00	-	< 50	47.6	< 5	105	< 0.625	< 10	52.0	< 1	< 1.6	< 6.3	< 1.3	< 30	1.42	22.68
2	115	4.95	-	3140	4720	561	123	< 3.125	< 50	173	< 5	< 8	< 31.5	< 6.5	< 150	24.1	< 12.5
3	-	0.01	-	80	10.1	< 5	19.7	< 0.625	< 10	263	< 1	< 1.6	< 6.3	1.90	< 30	3.73	< 2.5
4	-	0.54	-	< 5000	< 250	827	< 1250	< 62.5	< 1000	< 2000	< 100	< 160	< 630	< 130	< 3000	< 100	< 250
5	63.1	5.83	-	7261	710	618	89.0	5.85	< 10	20.3	< 1	< 1.6	< 6.3	< 1.3	< 30	13.7	< 2.5
6	-	2.44	93	479	24955	54.5	108	4.83	< 20	< 40	< 2	< 3.2	< 12.6	< 2.6	< 60	2.00	< 5
7	-	0.79	-	705	8154	104	37.9	10.1	< 10	< 20	< 1	< 1.6	< 6.3	< 1.3	30.0	4.58	< 2.5
8	-	4.18	-	3548	668	320	58.9	8.66	< 10	< 20	< 1	< 1.6	< 6.3	< 1.3	45.6	8.26	< 2.5
9	-	5.60	-	20782	227	3246	43.7	1.30	18.9	45.5	< 1	26.2	21.9	< 1.3	< 30	1.10	< 2.5
10	-	1.87	-	2029	38.0	1394	54.3	< 0.625	< 10	< 20	1.17	< 1.6	< 6.3	< 1.3	< 30	< 0.45	< 2.5
11	52.4	0.95	-	354	3587	461	115	< 3.125	< 50	< 100	< 5	< 8	< 31.5	< 6.5	< 150	12.2	< 12.5
12	-	0.64	-	252	3252	522	99.7	< 3.125	< 50	< 100	< 5	< 8	< 31.5	< 6.5	< 150	12.4	< 12.5
13	516	0.63	-	501	22907	322	1142	< 1.25	< 20	< 40	< 2	< 3.2	< 12.6	< 2.6	1331	52.6	< 5
14	-	5.94	-	9506	8387	4307	649	< 0.50	< 20	< 40	< 2	< 3.2	< 12.6	< 2.6	< 60	7.20	< 5

MRL – minimum reporting limit (ppb)
*Measured in the field using HACH portable spectrophotometer.
< denotes the value was below the limit of detection considering dilution factor

Supplementary Table 4. Dissolved gas composition (mol%) and calculated molar concentration based on Henry's Law for Peru flat-slab and back-arc hot springs

**Sp.**	**CO_2 mol %; mM**	**He mol %; µM**	**H_2 mol %; µM**	**Ar mol %; mM**	**O_2 mol %; mM**	**N_2 mol %; mM**	**CH_4 mol %; mM**	**CO mol %; mM**
2	51.16; 12.0	0.0386; 0.1522	0.1419; 1.0309	0.6099; 0.0066	8.917; 0.0826	39.14; 0.1966	0.0002; 0.0002	<0.0002
5	44.07; 9.1	0.0401; 0.1579	0.1659; 1.1703	0.6381; 0.0063	10.62; 0.0896	44.46; 0.2045	0.0002; 0.0002	<0.0002
6	66.59; 7.8	0.0508; 0.1994	0.1578; 0.9832	0.3155; 0.0021	5.040; 0.0286	27.18; 0.0860	0.6664; 0.4029	<0.0002
7	89.73; 15.1	0.0074; 0.0289	0.5689; 3.8374	0.1361; 0.0012	0.5348; 0.0039	8.840; 0.0355	0.1410; 0.1132	0.0450; 0.0003
8	96.95; 15.5	0.0028; 0.0111	0.0201; 0.035	0.0033; 0.000	0.4806; 0.0034	2.494; 0.0097	0.0453; 0.0350	<0.0002
9	20.88; 9.1	0.0015; 0.0058	0.0090; 0.0749	0.7372; 0.0123	17.60; 0.2525	60.77; 0.4604	0.0002; 0.0003	<0.0002
11	66.46; 6.5	0.1651; 0.6474	0.2486; 1.4864	0.2218; 0.0013	0.4391; 0.0022	32.39; 0.0905	0.0739; 0.0385	<0.0002
12	49.11; 6.1	0.1034; 0.4060	0.1838; 1.1585	0.4503; 0.0031	4.453; 0.0262	45.65; 0.1495	0.0539; 0.0340	<0.0002
13	78.59; 11.1	0.0067; 0.0263	0.0630; 0.4091	0.1771; 0.0013	0.6448; 0.0042	20.32; 0.0729	0.1960; 0.1374	<0.0002
14	90.06; 19.5	0.0093; 0.0368	0.2260; 1.6124	0.0896; 0.0009	0.2334; 0.0020	9.325; 0.0444	0.0600; 0.0589	<0.0002
Henry's Law Constant	3.4×10^{-4}	1.4×10^{-5}	7.8×10^{-6}	3.9×10^{-6}	1.4×10^{-5}	1.2×10^{-5}	6.4×10^{-6}	9.7×10^{-6}

Supplementary Table 5. Taxonomy for OTUs greater than ≥1% relative abundance in at least one of the 14 springs (n = 210).

OTU	Taxonomy	Phylum	Class	Order	Family	BLASTn Genus species	Accession
Otu00001	Bacteria(100)	Deinococcus-Thermus(100)	Deinococci(100)	Thermales(100)	Thermaceae(100)	Thermus sp. Tok20 A.1	L09665
Otu00002	Bacteria(100)	Deinococcus-Thermus(100)	Deinococci(100)	Thermales(100)	Thermaceae(100)	Meiothermus sp. Strain CFH 78305	MN826747
Otu00003	Bacteria(100)	Cyanobacteria/Chloroplast(100)	Cyanobacteria(100)	Family_I(100)	GpI(100)	Mastigocladus sp. CHP1	KX035101
Otu00004	Bacteria(100)	Chloroflexi(100)	Chloroflexia(100)	Chloroflexales(100)	Chloroflexaceae(100)	Chloroflexus aurantiacus partial	AJ308501
Otu00005	Bacteria(100)	Proteobacteria(100)	Zetaproteobacteria(100)	Mariprofundales(100)	Mariprofundaceae(100)	Mariprofundus ferrinatatus strain CP-8	CP018800
Otu00006	Bacteria(100)	Bacteroidetes(100)	Bacteroidetes_unclassified(100)	Bacteroidetes_unclassified(100)	Bacteroidetes_unclassified(100)	Cytophaga sp. T2/21.8	HQ658664
Otu00007	Bacteria(100)	Firmicutes(100)	Bacilli(100)	Bacillales(100)	Bacillaceae_1(95)	Bacillus thioparans strain 1B12	MH929667
Otu00008	Archaea(100)	Crenarchaeota(100)	Thermoprotei(100)	Desulfurococcales(100)	Desulfurococcaceae(100)	Thermodiscus maritimus strain S2	NR_044907
Otu00009	Bacteria(100)	Firmicutes(100)	Clostridia(100)	Clostridiales(100)	Clostridiaceae_1(100)	Clostridium favososporum	JQ897403
Otu00010	Bacteria(100)	Bacteroidetes(100)	Sphingobacteriia(100)	Sphingobacteriales(100)	Cyclobacteriaceae(100)	Algoriphagus taiwanensis strain CC-PR-82	NR_148276
Otu00011	Bacteria(100)	Cyanobacteria/Chloroplast(100)	Chloroplast(100)	Chloroplast(100)	Bacillariophyta(99)	Vaucheria litorea chloroplast	EU912438
Otu00012	Archaea(100)	Thaumarchaeota(100)	Nitrososphaerales(100)	Nitrososphaeraceae(100)	Nitrososphaera(100)	Candidatus Nitrosocosmicus sp. Kfb	KX863712
Otu00013	Bacteria(100)	Bacteria_unclassified(100)	Bacteria_unclassified(100)	Bacteria_unclassified(100)	Bacteria_unclassified(100)	Thermodesulfovibrio yellowstonii DSM 11347	CP001147
Otu00014	Bacteria(100)	Proteobacteria(100)	Gammaproteobacteria(100)	Vibrionales(100)	Vibrionaceae(100)	Vibrio sp. strain 201707CJKOP-Y195	MG593759
Otu00015	Bacteria(100)	Firmicutes(100)	Bacilli(100)	Bacillales(100)	Planococcaceae(83)	Bacillus halmapalus strain DSM 8723	NR_026144
Otu00016	Bacteria(100)	Bacteria_unclassified(100)	Bacteria_unclassified(100)	Bacteria_unclassified(100)	Bacteria_unclassified(100)	Filamentous bacterium EU25	DQ232757
Otu00017	Bacteria(100)	Cyanobacteria/Chloroplast(99)	Cyanobacteria(99)	Cyanobacteria_unclassified(99)	Cyanobacteria_unclassified(99)	Cyanobacterium aponinum Kh.A	MH179053
Otu00018	Bacteria(100)	Proteobacteria(100)	Gammaproteobacteria(100)	Xanthomonadales(100)	Xanthomonadaceae(100)	Pseudoxanthomonas mexicana strain EGE-B-3	KP050791
Otu00019	Archaea(100)	Archaea_unclassified(100)	Archaea_unclassified(100)	Archaea_unclassified(100)	Archaea_unclassified(100)	Methanogenic archaeon CH50	DQ513419
Otu00020	Bacteria(100)	Bacteroidetes(100)	Flavobacteriia(100)	Flavobacteriales(100)	Flavobacteriaceae(100)	Aureisphaera salina strain A6D-50	NR_151885
Otu00021	Bacteria(100)	Firmicutes(100)	Bacilli(100)	Bacillales(100)	Bacillaceae_1(97)	Bacillus sp. Y1 chromosome	CP030028
Otu00022	Bacteria(100)	Cyanobacteria/Chloroplast(100)	Chloroplast(100)	Chloroplast(100)	Bacillariophyta(100)	Odontella sinensis	Z67753
Otu00023	Bacteria(100)	Cyanobacteria/Chloroplast(100)	Cyanobacteria(100)	Cyanobacteria_unclassified(100)	Cyanobacteria_unclassified(100)	Pseudanabaenaceae cyanobacterium CENA319	KT731140
Otu00024	Archaea(100)	Archaea_unclassified(100)	Archaea_unclassified(100)	Archaea_unclassified(100)	Archaea_unclassified(100)	Methanogenic archaeon CH50	DQ513419

OTU	Taxonomy	Phylum	Class	Order	Family	BLASTn Genus species	Accession
Otu00025	Bacteria(100)	Proteobacteria(100)	Betaproteobacteria(100)	Burkholderiales(100)	Comamonadaceae(99)	Hydrogenophaga aquatica strain CC-KL-3	NR_158120
Otu00026	Bacteria(100)	Firmicutes(100)	Clostridia(100)	Clostridiales(100)	Clostridiaceae_1(100)	Clostridium sp. BG-C66	FJ384378
Otu00027	Bacteria(100)	Chloroflexi(100)	Chloroflexia(100)	Chloroflexales(100)	Oscillochloridaceae(95)	Candidatus Chloranaerofilum corporosum clone JGI24185J35167_10218671	KY937209
Otu00028	Bacteria(100)	Chloroflexi(100)	Chloroflexia(100)	Chloroflexales(100)	Chloroflexaceae(100)	Roseiflexus castenholzii strain HLO8	NR_112114
Otu00029	Bacteria(100)	Firmicutes(100)	Bacilli(100)	Bacillales(100)	Bacillales_Incertae_Sedis_XII(100)	Exiguobacterium mexicanum strain 0A641	MH929903
Otu00030	Bacteria(100)	Deinococcus-Thermus(100)	Deinococci(100)	Thermales(100)	Thermaceae(100)	Thermus kawarayensis strain KW11	NR_112160
Otu00031	Bacteria(100)	Deinococcus-Thermus(99)	Deinococci(99)	Thermales(99)	Thermaceae(99)	Thermus Tok3 A.1	L10069
Otu00032	Bacteria(100)	Chloroflexi(100)	Chloroflexi_unclassified(97)	Chloroflexi_unclassified(97)	Chloroflexi_unclassified(97)	Longilinea arvoryzae strain KOME-1	NR_041355
Otu00033	Bacteria(100)	Proteobacteria(100)	Alphaproteobacteria(100)	Rhodobacterales(100)	Rhodobacteraceae(100)	Sediminimonas qiaohouensis strain YIM B025	EU878004
Otu00034	Bacteria(100)	Proteobacteria(100)	Gammaproteobacteria(100)	Chromatiales(100)	Chromatiaceae(100)	Rheinheimera sp. F8	CP013656
Otu00035	Bacteria(100)	Proteobacteria(100)	Betaproteobacteria(100)	Rhodocyclales(99)	Rhodocyclaceae(99)	Methyloversatilis discipulorum strain B4	MG757549
Otu00036	Bacteria(100)	Proteobacteria(100)	Betaproteobacteria(100)	Gallionellales(99)	Gallionellaceae(99)	Sideroxydans paludicola strain BrT	DQ386858
Otu00037	Bacteria(100)	Firmicutes(100)	Bacilli(100)	Bacillales(100)	Bacillales_unclassified(100)	Paenibacillus sp. D-1	KF020729
Otu00038	Bacteria(100)	Firmicutes(100)	Clostridia(100)	Clostridiales(100)	Clostridiaceae_1(100)	Clostridium quinii strain DSM 6736	NR_026149
Otu00039	Bacteria(100)	Bacteroidetes(100)	Bacteroidetes_unclassified(100)	Bacteroidetes_unclassified(100)	Bacteroidetes_unclassified(100)	Cytophaga sp. T2/21.8	HQ658664
Otu00040	Bacteria(100)	Proteobacteria(100)	Gammaproteobacteria(100)	Alteromonadales(100)	Shewanellaceae(100)	Shewanella sp. strain L8-6-3	MG561188
Otu00041	Archaea(100)	Thaumarchaeota(100)	Nitrososphaerales(100)	Nitrososphaeraceae(100)	Nitrososphaera(100)	Octopus Spring nitrifying crenarchaeote OS70	EU239962
Otu00042	Bacteria(100)	Proteobacteria(100)	Gammaproteobacteria(100)	Chromatiales(100)	Chromatiaceae(100)	Rheinheimera japonica	LC011485
Otu00043	Bacteria(100)	Spirochaetes(100)	Spirochaetia(100)	Spirochaetales(100)	Spirochaetaceae(100)	Oceanispirochaeta sediminicola strain SY2	NR_158023
Otu00044	Bacteria(100)	Proteobacteria(100)	Alphaproteobacteria(100)	Rhodobacterales(100)	Rhodobacteraceae(100)	Rhodobacter sp. KT6	FJ439600
Otu00045	Bacteria(100)	Spirochaetes(100)	Spirochaetia(100)	Spirochaetales(100)	Spirochaetaceae(100)	Spirochaeta perfilievii strain P	NR_115202
Otu00046	Bacteria(100)	Bacteroidetes(100)	Sphingobacteriia(100)	Sphingobacteriales(100)	Rhodothermaceae(100)	Rhodothermus marinus strain R-10	NR_029282
Otu00047	Bacteria(100)	Bacteria_unclassified(100)	Bacteria_unclassified(100)	Bacteria_unclassified(100)	Bacteria_unclassified(100)	Candidatus Magnetoovum mohavensis strain LO-1	GU979422
Otu00048	Bacteria(100)	Proteobacteria(100)	Betaproteobacteria(100)	Betaproteobacteria_unclassified(100)	Betaproteobacteria_unclassified(100)	Bacterium SCGC AAA018-M4	HQ290506

OTU	Taxonomy	Phylum	Class	Order	Family	BLASTn Genus species	Accession
Otu00049	Bacteria(100)	Proteobacteria(100)	Gammaproteobacteria(100)	Vibrionales(100)	Vibrionaceae(100)	Vibrio ruber strain HMF8004	KY047409
Otu00050	Bacteria(100)	Bacteroidetes(93)	Bacteroidetes_unclassified(93)	Bacteroidetes_unclassified(93)	Bacteroidetes_unclassified(93)	Pedobacter quisquiliarum strain C62-2	KU973598
Otu00051	Bacteria(100)	Bacteria_unclassified(100)	Bacteria_unclassified(100)	Bacteria_unclassified(100)	Bacteria_unclassified(100)	Nitrospira sp. SRI-9	AF255603
Otu00052	Bacteria(100)	Chloroflexi(100)	Anaerolineae(100)	Anaerolineales(100)	Anaerolineaceae(100)	Thermomarinilinea lacunifontana strain SW7	NR_132293
Otu00053	Bacteria(100)	Proteobacteria(100)	Betaproteobacteria(100)	Burkholderiales(100)	Burkholderiaceae(95)	Limnobacter thiooxidans strain AD12	KY284082
Otu00054	Bacteria(100)	Proteobacteria(100)	Alphaproteobacteria(100)	Rhodobacterales(100)	Rhodobacteraceae(100)	Pseudoruegeria sp. R-52653	KT185147
Otu00055	Bacteria(100)	Proteobacteria(100)	Betaproteobacteria(100)	Betaproteobacteria_unclassified(100)	Betaproteobacteria_unclassified(100)	Candidatus Nitrotoga sp. AM1	LC190436
Otu00056	Bacteria(100)	Bacteroidetes(100)	Sphingobacteriia(100)	Sphingobacteriales(100)	Chitinophagaceae(100)	Sediminibacterium goheungense strain HME7863	NR_133854
Otu00057	Bacteria(100)	Firmicutes(100)	Bacilli(100)	Bacillales(100)	Bacillales_unclassified(91)	Bacillus luteolus strain YIM 93174	NR_108638
Otu00058	Bacteria(99)	Bacteria_unclassified(99)	Bacteria_unclassified(99)	Bacteria_unclassified(99)	Bacteria_unclassified(99)	Limibaculum halophilum strain CAU 1123	NR_158129
Otu00059	Bacteria(100)	Proteobacteria(100)	Alphaproteobacteria(100)	Rhodobacterales(100)	Rhodobacteraceae(100)	Haematobacter massiliensis strain KC2145	NR_115743
Otu00060	Bacteria(100)	Bacteria_unclassified(100)	Bacteria_unclassified(100)	Bacteria_unclassified(100)	Bacteria_unclassified(100)	Chloroflexi bacterium Ver9Iso1	DQ812549
Otu00061	Bacteria(100)	Bacteria_unclassified(100)	Bacteria_unclassified(100)	Bacteria_unclassified(100)	Bacteria_unclassified(100)	Candidatus Roseilinea gracile clone JGI24185J35167_10016968	KY937207
Otu00062	Bacteria(100)	Proteobacteria(100)	Gammaproteobacteria(100)	Gammaproteobacteria_incertae_sedis(100)	Solimonas(100)	Sinimarinibacterium flocculans strain NH6-24	NR_137419
Otu00063	Archaea(100)	Archaea_unclassified(100)	Archaea_unclassified(100)	Archaea_unclassified(100)	Archaea_unclassified(100)	Methanogenic archaeon CH50	DQ513419
Otu00064	Bacteria(100)	Bacteroidetes(100)	Sphingobacteriia(100)	Sphingobacteriales(100)	Rhodothermaceae(100)	Rhodothermus marinus strain R-10	NR_029282
Otu00065	Bacteria(100)	Proteobacteria(100)	Alphaproteobacteria(100)	Rhodobacterales(100)	Rhodobacteraceae(100)	Phaeobacter sp. URN3	AB916871
Otu00066	Bacteria(100)	Proteobacteria(100)	Betaproteobacteria(100)	Betaproteobacteria_unclassified(100)	Betaproteobacteria_unclassified(100)	Bacterium B10D1	JX869434
Otu00067	Bacteria(100)	Bacteroidetes(100)	Bacteroidetes_unclassified(100)	Bacteroidetes_unclassified(100)	Bacteroidetes_unclassified(100)	Lentimicrobium saccharophilum strain TBC1	NR_149795
Otu00068	Bacteria(100)	Cyanobacteria/Chloroplast(100)	Cyanobacteria(100)	Cyanobacteria_unclassified(100)	Cyanobacteria_unclassified(100)	Oscillatoria amphigranulata strain 11-3	AF317503
Otu00069	Bacteria(100)	Bacteria_unclassified(100)	Bacteria_unclassified(100)	Bacteria_unclassified(100)	Bacteria_unclassified(100)	Candidatus Acetothermus autotrophicum	AP011801
Otu00070	Archaea(100)	Archaea_unclassified(100)	Archaea_unclassified(100)	Archaea_unclassified(100)	Archaea_unclassified(100)	Methanospirillum sp.	L48407
Otu00071	Bacteria(100)	Proteobacteria(100)	Gammaproteobacteria(100)	Alteromonadales(100)	Alteromonadaceae(100)	Alteromonas aestuariivivens strain JDTF-113	NR_157790
Otu00072	Bacteria(100)	Bacteria_unclassified(100)	Bacteria_unclassified(100)	Bacteria_unclassified(100)	Bacteria_unclassified(100)	Ornatilinea apprima strain P3M-1	NR_109544

OTU	Taxonomy	Phylum	Class	Order	Family	BLASTn Genus species	Accession
Otu00073	Bacteria(100)	Bacteria_unclassified(100)	Bacteria_unclassified(100)	Bacteria_unclassified (100)	Bacteria_unclassified(100)	Thermoanaerobaculum aquaticum strain MP-01	NR_109681
Otu00074	Bacteria(100)	Proteobacteria(100)	Gammaproteobacteria(100)	Pseudomonadales(100)	Pseudomonadaceae(100)	Pseudomonas sp. strain B-BETUL-M	MG664256
Otu00075	Bacteria(100)	Ignavibacteriae(100)	Ignavibacteria(100)	Ignavibacteriales(100)	Ignavibacteriaceae(100)	Ignavibacterium album strain JCM 16511	NR_074698
Otu00076	Bacteria(100)	Proteobacteria(100)	Betaproteobacteria(100)	Rhodocyclales(98)	Rhodocyclaceae(98)	Thauera sp. MZ1T	CP001281
Otu00077	Archaea(100)	Crenarchaeota(98)	Thermoprotei(98)	Thermoprotei_unclassified(98)	Thermoprotei_unclassified(98)	Thermofilum uzonense strain 1807-2	NR_146002
Otu00078	unknown(100)	unknown_unclassified(100)	unknown_unclassified(100)	unknown_unclassified(100)	unknown_unclassified(100)	unknown_unclassified(100)	
Otu00079	Bacteria(100)	Proteobacteria(100)	Gammaproteobacteria(100)	Aeromonadales(100)	Aeromonadaceae(100)	Aeromonas caviae strain R25-2	CP025777
Otu00080	Bacteria(100)	Bacteroidetes(100)	Bacteroidetes_unclassified(100)	Bacteroidetes_unclassified(100)	Bacteroidetes_unclassified(100)	Lewinella xylanilytica strain 13-9-B8	NR_137393
Otu00081	Bacteria(100)	Bacteroidetes(100)	Bacteroidetes_unclassified(100)	Bacteroidetes_unclassified(100)	Bacteroidetes_unclassified(100)	Bacterium SCGC AAA018-K8	HQ290503
Otu00082	Bacteria(100)	Proteobacteria(100)	Zetaproteobacteria(100)	Mariprofundales(100)	Mariprofundaceae(100)	Mariprofundus micogutta	LC107871
Otu00083	Bacteria(100)	candidate_division_WPS-2(100)	candidate_division_WPS-2_unclassified(100)	candidate_division_WPS-2_unclassified(100)	candidate_division_WPS-2_unclassified(100)	Tepidisphaera mucosa strain 2813	KM052380
Otu00084	Bacteria(100)	Proteobacteria(100)	Alphaproteobacteria (100)	Sphingomonadales(100)	Sphingomonadaceae(100)	Alpha proteobacterium Mzj1	AF531016
Otu00085	Bacteria(100)	Proteobacteria(100)	Gammaproteobacteria(100)	Alteromonadales(100)	Alteromonadaceae(100)	Agarivorans gilvus strain WH0801	NR_117238
Otu00086	Bacteria(100)	Proteobacteria(100)	Betaproteobacteria(100)	Burkholderiales(100)	Comamonadaceae(98)	Comamonadaceae bacterium strain hainich_106	MG980500
Otu00087	Bacteria(100)	Proteobacteria(100)	Betaproteobacteria(100)	Burkholderiales(100)	Comamonadaceae(100)	Rhodoferax sp. strain BDP12	MG712812
Otu00088	Bacteria(100)	Bacteroidetes(100)	Bacteroidetes_unclassified(100)	Bacteroidetes_unclassified(100)	Bacteroidetes_unclassified(100)	Fluviicola hefeinensis strain MYL-8	NR_133750
Otu00089	Bacteria(100)	Bacteria_unclassified(100)	Bacteria_unclassified(100)	Bacteria_unclassified (100)	Bacteria_unclassified(100)	Candidatus Kaiserbacteria bacterium GW2011_GWA2_52_12	KX123441
Otu00090	Bacteria(100)	Proteobacteria(100)	Gammaproteobacteria(100)	Chromatiales(100)	Chromatiaceae(100)	Rheinheimera sp. WMF-1	AM690025
Otu00091	Bacteria(100)	Bacteroidetes(100)	Sphingobacteriia(100)	Sphingobacteriales(100)	Chitinophagaceae(100)	Sediminibacterium salmoneum strain NJ-44	NR_044197
Otu00092	Bacteria(100)	Proteobacteria(100)	Betaproteobacteria(100)	Burkholderiales(100)	Comamonadaceae(100)	Hydrogenophaga taeniospiralis strain C2PO1	JQ689193
Otu00093	Bacteria(100)	Proteobacteria(100)	Gammaproteobacteria(100)	Xanthomonadales(100)	Xanthomonadaceae(100)	Lysobacter hankyongensis strain KTce-2	NR_147746
Otu00094	Bacteria(100)	Bacteroidetes(100)	Flavobacteriia(100)	Flavobacteriales(100)	Flavobacteriaceae(100)	Aureisphaera salina strain A6D-50	NR_151885
Otu00095	Bacteria(100)	Proteobacteria(100)	Gammaproteobacteria(100)	Pseudomonadales(100)	Pseudomonadaceae(100)	Pseudomonadaceae bacterium strain hainich_182	MG980551

OTU	Taxonomy	Phylum	Class	Order	Family	BLASTn Genus species	Accession
Otu00096	Bacteria(100)	Proteobacteria(100)	Gammaproteobacteria(100)	Chromatiales(100)	Chromatiaceae(100)	Rheinheimera sp. A1a-3	HG738873
Otu00097	Bacteria(100)	Acidobacteria(100)	Acidobacteria_Gp3(100)	Gp3(95)	Gp3_unclassified(95)	Paludibaculum fermentans strain P105	NR_134120
Otu00098	Bacteria(100)	Bacteroidetes(100)	Flavobacteriia(100)	Flavobacteriales(100)	Flavobacteriaceae(100)	Flavobacterium gelidilacus	AJ871468
Otu00099	Bacteria(100)	Chloroflexi(100)	Anaerolineae(100)	Anaerolineales(100)	Anaerolineaceae(100)	Anaerolinea thermophila strain UNI-1	NR_036818
Otu00100	Bacteria(100)	Proteobacteria(100)	Gammaproteobacteria(100)	Chromatiales(100)	Chromatiaceae(100)	Rheinheimera sp. 235	JQ012975
Otu00101	Bacteria(100)	Proteobacteria(100)	Alphaproteobacteria(100)	Sphingomonadales(100)	Sphingomonadaceae(100)	Sphingomonas asaccharolytica strain MadaFrogSkinBac.DB-.4184	MF526480
Otu00102	Bacteria(100)	Bacteroidetes(100)	Flavobacteriia(100)	Flavobacteriales(100)	Cryomorphaceae(100)	Bacterium SCGC AAA166-P03	JF488636
Otu00103	Archaea(100)	Thaumarchaeota(100)	Nitrososphaerales(100)	Nitrososphaeraceae(100)	Nitrososphaera(100)	Thaumarchaeota archaeon C13	KU290365
Otu00104	Bacteria(100)	Bacteria_unclassified(100)	Bacteria_unclassified(100)	Bacteria_unclassified(100)	Bacteria_unclassified(100)	Gram-positive bacterial sp. (strain Ok9. B1)	L09186
Otu00105	Bacteria(100)	Proteobacteria(100)	Betaproteobacteria(100)	Burkholderiales(100)	Burkholderiales_unclassified(100)	Comamonadaceae bacterium PIV-3D	AJ505855
Otu00106	Bacteria(100)	Proteobacteria(100)	Proteobacteria_unclassified(100)	Proteobacteria_unclassified(100)	Proteobacteria_unclassified(100)	Candidatus Combothrix italica clone BIO53	AY590699
Otu00107	Bacteria(100)	Cyanobacteria/Chloroplast(100)	Chloroplast(100)	Chloroplast(100)	Bacillariophyta(100)	Synedra acus chloroplast	JQ088178
Otu00108	Bacteria(100)	Firmicutes(100)	Clostridia(100)	Clostridiales(100)	Clostridiales_unclassified(98)	Anaerosolibacter carboniphilus strain IRF19	KF601947
Otu00109	Bacteria(100)	Cyanobacteria/Chloroplast(100)	Cyanobacteria(100)	Cyanobacteria_unclassified(99)	Cyanobacteria_unclassified(99)	Planktothrix sp. CYN61	GQ853411
Otu00110	Bacteria(100)	Proteobacteria(100)	Alphaproteobacteria(100)	Rhodospirillales(100)	Rhodospirillaceae(100)	Azospirillum sp. NS33	AB480702
Otu00111	Bacteria(100)	Cyanobacteria/Chloroplast(99)	Cyanobacteria(89)	Cyanobacteria_unclassified(89)	Cyanobacteria_unclassified(89)	Geminocystis sp. NIES-3709	AP014821
Otu00112	Bacteria(100)	Chloroflexi(100)	Chloroflexi_unclassified(100)	Chloroflexi_unclassified(100)	Chloroflexi_unclassified(100)	Bellilinea caldifistulae strain GOMI-1	NR_041354
Otu00113	Bacteria(100)	Bacteria_unclassified(100)	Bacteria_unclassified(100)	Bacteria_unclassified(100)	Bacteria_unclassified(100)	Maledivibacter halophilus strain DSM 5387	NR_125713
Otu00114	Bacteria(100)	Cyanobacteria/Chloroplast(100)	Cyanobacteria(100)	Cyanobacteria_unclassified(100)	Cyanobacteria_unclassified(100)	Geitlerinema sp. Pond 63 16S	KJ140095
Otu00115	Archaea(100)	Archaea_unclassified(100)	Archaea_unclassified(100)	Archaea_unclassified(100)	Archaea_unclassified(100)	Methanospirillum sp.	L48407
Otu00116	Bacteria(100)	Proteobacteria(100)	Proteobacteria_unclassified(100)	Proteobacteria_unclassified(100)	Proteobacteria_unclassified(100)	Anaeromyxobacter dehalogenans strain FRC-W	FJ190062
Otu00117	Bacteria(100)	Bacteria_unclassified(100)	Bacteria_unclassified(100)	Bacteria_unclassified(100)	Bacteria_unclassified(100)	Candidatus Roseilinea gracile clone JGI24185J35167_10016968	KY937207
Otu00118	Bacteria(100)	Bacteroidetes(100)	Flavobacteriia(100)	Flavobacteriales(100)	Flavobacteriaceae(100)	Psychroflexus saliphilus strain WDS4A13	NR_153694
Otu00119	Bacteria(100)	Bacteria_unclassified(100)	Bacteria_unclassified(100)	Bacteria_unclassified(100)	Bacteria_unclassified(100)	Filamentous bacterium EU25	DQ232757

OTU	Taxonomy	Phylum	Class	Order	Family	BLASTn Genus species	Accession
Otu00120	Bacteria(100)	Proteobacteria(100)	Betaproteobacteria(100)	Burkholderiales(100)	Comamonadaceae(99)	Hydrogenophaga sp. PBC	CP017311
Otu00121	Bacteria(100)	Bacteria_unclassified(100)	Bacteria_unclassified(100)	Bacteria_unclassified (100)	Thaumarchaeota_unclassified (100)	Candidatus Nitrosotalea devanaterra isolate Nd1	JN227488
Otu00122	Bacteria(100)	Bacteroidetes(100)	Flavobacteriia(100)	Flavobacteriales(100)	Flavobacteriaceae(100)	Lutibacter maritimus strain S7-2	NR_116738
Otu00123	Bacteria(100)	Proteobacteria(100)	Gammaproteobacteria(100)	Pseudomonadales(100)	Pseudomonadaceae(100)	Cellvibrio fontiphilus strain MVW-40	NR_157755
Otu00124	Bacteria(100)	Bacteroidetes(100)	Bacteroidetes_unclassified(100)	Bacteroidetes_unclassified(100)	Bacteroidetes_unclassified(100)	Lentimicrobium saccharophilum strain TBC1	NR_149795
Otu00125	Bacteria(100)	Proteobacteria(100)	Deltaproteobacteria (100)	Desulfuromonadales(100)	Desulfuromonadaceae(100)	Desulfuromusa ferrireducens strain 102	NR_043214
Otu00126	Archaea(100)	Archaea_unclassified(100)	Archaea_unclassified(100)	Archaea_unclassified (100)	Archaea_unclassified(100)	Methanogenic archaeon CH50	DQ513419
Otu00127	Bacteria(100)	Proteobacteria(100)	Alphaproteobacteria (100)	Alphaproteobacteria_unclassified(98)	Vibrionaceae(100)	Vibrio anguillarum strain AL8-6W	MH881409
Otu00128	Bacteria(100)	Bacteria_unclassified(100)	Bacteria_unclassified(100)	Bacteria_unclassified (100)	Bacteria_unclassified(100)	Kroppenstedtia sanguinis strain X0394	NR_146671
Otu00129	Bacteria(100)	Proteobacteria(100)	Deltaproteobacteria (100)	Desulfuromonadales(100)	Desulfuromonadales_unclassified(97)	Delta Proteobacterium G50VI	AJ786070
Otu00130	Bacteria(100)	Cyanobacteria/Chloroplast(100)	Cyanobacteria(100)	Cyanobacteria_unclassified(100)	Cyanobacteria_unclassified(100)	Microcoleus chthonoplastes CCY9603	GQ402020
Otu00131	Bacteria(100)	Proteobacteria(100)	Alphaproteobacteria (100)	Rhodobacterales(100)	Rhodobacteraceae(100)	Paracoccus koreensis strain Ch05	NR_041238
Otu00132	Bacteria(100)	Proteobacteria(100)	Deltaproteobacteria (96)	Bdellovibrionales(96)	Bacteriovoracaceae(96)	Bacteriovorax sp. MER21	DQ631740
Otu00133	Bacteria(100)	Bacteroidetes(100)	Flavobacteriia(100)	Flavobacteriales(100)	Flavobacteriaceae(100)	Flavobacterium sp. strain N72	MH714638
Otu00134	Bacteria(100)	Firmicutes(100)	Clostridia(100)	Clostridiales(100)	Lachnospiraceae(100)	Cellulosilyticum lentocellum strain DSM 5427	NR_119067
Otu00135	Bacteria(100)	Proteobacteria(100)	Alphaproteobacteria (100)	Rhodospirillales(100)	Rhodospirillaceae(98)	Elstera cyanobacteriorum strain TH019	NR_158149
Otu00136	Bacteria(100)	Bacteria_unclassified(99)	Bacteria_unclassified(99)	Bacteria_unclassified (99)	Bacteria_unclassified(99)	Olavius ilvae Delta 1 endosymbiont	AJ620500
Otu00137	Bacteria(100)	Bacteria_unclassified(100)	Bacteria_unclassified(100)	Bacteria_unclassified (100)	Bacteria_unclassified(100)	Melioribacter roseus strain P3M-2	NR_074796
Otu00138	Bacteria(100)	Proteobacteria(100)	Gammaproteobacteria(100)	Xanthomonadales(100)	Xanthomonadaceae(100)	Xanthomonadaceae bacterium strain hainich_160	MG980541
Otu00139	Bacteria(100)	Bacteria_unclassified(100)	Bacteria_unclassified(100)	Bacteria_unclassified (100)	Bacteria_unclassified(100)	Coxiella endosymbiont of Rhipicephalus microplus isolate Rhmicro2	KP994840
Otu00141	Bacteria(100)	Cyanobacteria/Chloroplast(100)	Cyanobacteria(100)	Cyanobacteria_unclassified(100)	Cyanobacteria_unclassified(100)	Desertifilum dzianense PMC 909.15	MF579907
Otu00144	Bacteria(100)	Bacteroidetes(100)	Bacteroidetes_unclassified(100)	Bacteroidetes_unclassified(100)	Bacteroidetes_unclassified(100)	Bacterium strain BB12	KX815444
Otu00145	Bacteria(100)	Armatimonadetes(100)	Armatimonadetes_gp7(100)	Armatimonadetes_gp7_unclassified(100)	Armatimonadetes_gp7_unclassified(100)	Eubacterium sp. (OS type L)	L04707
Otu00146	Bacteria(100)	Firmicutes(100)	Clostridia(100)	Clostridiales(100)	Clostridiales_unclassified(100)	Abyssivirga alkaniphila strain L81	NR_148837

OTU	Taxonomy	Phylum	Class	Order	Family	BLASTn Genus species	Accession
Otu00147	Bacteria(100)	Proteobacteria(100)	Alphaproteobacteria (100)	Rhodobacterales(100)	Rhodobacteraceae(100)	Gemmobacter sp. strain 3AM-11	KT992325
Otu00149	Archaea(100)	Thaumarchaeota(100)	Thaumarchaeota_unclassified(100)	Thaumarchaeota_unclassified(100)	Bacteria_unclassified(100)	Ignavibacterium album strain JCM 16511	NR_074698
Otu00150	Bacteria(100)	Bacteroidetes(99)	Bacteroidetes_unclassified(99)	Bacteroidetes_unclassified(99)	Bacteroidetes_unclassified(99)	Bacteroides sp. ECP-C1	AF529225
Otu00151	Archaea(100)	Archaea_unclassified(100)	Archaea_unclassified(100)	Archaea_unclassified (100)	Archaea_unclassified(100)	Palaeococcus helgesonii strain PI1	NR_029059
Otu00152	Bacteria(100)	Proteobacteria(100)	Gammaproteobacteria(100)	Gammaproteobacteria_incertae_sedis(100)	Solimonas(100)	Sinimarinibacterium flocculans strain NH6-24	NR_137419
Otu00153	Bacteria(100)	Bacteroidetes(100)	Bacteroidetes_unclassified(100)	Bacteroidetes_unclassified(100)	Bacteroidetes_unclassified(100)	Bacteroidetes bacterium PPf50E2	AY548787
Otu00155	Bacteria(100)	Proteobacteria(100)	Gammaproteobacteria(100)	Pseudomonadales(100)	Moraxellaceae(100)	Perlucidibaca aquatica strain BK296	NR_157660
Otu00158	Bacteria(100)	Bacteroidetes(100)	Flavobacteriia(100)	Flavobacteriales(100)	Flavobacteriaceae(100)	F.aquatile	M62797 M28236
Otu00159	Bacteria(100)	Proteobacteria(100)	Gammaproteobacteria(100)	Alteromonadales(100)	Alteromonadaceae(100)	Marinobacter hydrocarbonoclasticus strain E13	MH746060
Otu00160	Archaea(100)	Thaumarchaeota(100)	Nitrososphaerales(100)	Nitrososphaeraceae(100)	Nitrososphaera(100)	Thaumarchaeota archaeon C13	KU290365
Otu00161	Bacteria(100)	Proteobacteria(100)	Proteobacteria_unclassified(100)	Proteobacteria_unclassified(100)	Proteobacteria_unclassified(100)	Desulfomicrobium macestii strain M-9	NR_025349
Otu00162	Bacteria(100)	Bacteroidetes(100)	Bacteroidetes_incertae_sedis(100)	Ohtaekwangia(100)	Ohtaekwangia_unclassified(100)	Bacteroidetes bacterium KJY	AB540000
Otu00163	Bacteria(100)	Proteobacteria(100)	Alphaproteobacteria (100)	Rhodobacterales(100)	Rhodobacteraceae(100)	Rubrimonas shengliensis strain SL014B-80A	GU125652
Otu00164	Bacteria(100)	Bacteroidetes(99)	Bacteroidetes_unclassified(99)	Bacteroidetes_unclassified(99)	Bacteroidetes_unclassified(99)	Mucilaginibacter pedocola strain TBZ30	NR_152040
Otu00165	Bacteria(100)	Bacteria_unclassified(100)	Bacteria_unclassified(100)	Bacteria_unclassified (100)	Bacteria_unclassified(100)	Desulfonatronum thioautotrophicum strain ASO4-1	NR_116693
Otu00166	Bacteria(100)	Bacteroidetes(100)	Flavobacteriia(100)	Flavobacteriales(100)	Flavobacteriaceae(100)	Mariniflexile jejuense strain SSK2-3	NR_109619
Otu00168	Bacteria(100)	Proteobacteria(100)	Gammaproteobacteria(100)	Vibrionales(100)	Alphaproteobacteria_unclassified(98)	Pannonibacter sp. VTT E-073067	EU438959
Otu00169	Bacteria(100)	Proteobacteria(100)	Gammaproteobacteria(100)	Xanthomonadales(100)	Xanthomonadaceae(100)	Arenimonas daejeonensis strain T7-07	NR_108139
Otu00170	Archaea(100)	Archaea_unclassified(100)	Archaea_unclassified(100)	Archaea_unclassified (100)	Archaea_unclassified(100)	Candidatus Caldiarchaeum subterraneum	AP011878
Otu00173	Bacteria(100)	Proteobacteria(100)	Gammaproteobacteria(100)	Gammaproteobacteria_incertae_sedis(100)	Solimonas(100)	Solimonas flava strain CW-KD 4	NR_044123
Otu00174	Bacteria(100)	Bacteroidetes(100)	Sphingobacteriia(94)	Sphingobacteriales(94)	Sphingobacteriales_unclassified(94)	Candidatus Aquirestis calciphila	AJ786331
Otu00175	Bacteria(100)	Bacteroidetes(100)	Flavobacteriia(100)	Flavobacteriales(100)	Flavobacteriaceae(100)	Flavobacterium inkyongense strain IMCC27201	NR_156036
Otu00177	Bacteria(100)	Chloroflexi(96)	Chloroflexi_unclassified(96)	Chloroflexi_unclassified(96)	Chloroflexi_unclassified(96)	Thermomarinilinea lacunifontana strain SW7	NR_132293
Otu00179	Bacteria(100)	Proteobacteria(100)	Gammaproteobacteria(100)	Pseudomonadales(100)	Pseudomonadaceae(100)	Cellvibrio fulvus strain NCIMB 8634	NR_025210

OTU	Taxonomy	Phylum	Class	Order	Family	BLASTn Genus species	Accession
Otu00180	Bacteria(100)	Proteobacteria(100)	Deltaproteobacteria (100)	Desulfuromonadales(100)	Desulfuromonadaceae(97)	Delta proteobacterium EtOHpelo	AY771935
Otu00181	Bacteria(100)	Proteobacteria(99)	Deltaproteobacteria (92)	Bdellovibrionales(90)	Bacteriovoracaceae(90)	Bacteriovorax sp. MER21	DQ631740
Otu00182	Archaea(100)	Thaumarchaeota(10 0)	Nitrososphaerales(9 6)	Nitrososphaeraceae(9 6)	Gp23_unclassified(100)	Eubacterium sp. (OS type K)	L04711
Otu00183	Bacteria(100)	Bacteroidetes(100)	Bacteroidetes_uncla ssified(100)	Bacteroidetes_unclas sified(100)	Bacteroidetes_unclassified(10 0)	Bacteroidetes bacterium SCGC AAA487-A14	HQ663650
Otu00184	Bacteria(100)	Proteobacteria(100)	Proteobacteria_uncl assified(95)	Proteobacteria_uncla ssified(95)	Proteobacteria_unclassified(9 5)	Marine alpha proteobacterium RS.Sph.017	DQ097291
Otu00185	Bacteria(100)	Proteobacteria(100)	Gammaproteobacter ia(100)	Xanthomonadales(10 0)	Xanthomonadaceae(100)	Thermomonas sp. ROi19	EF219041
Otu00186	Bacteria(100)	Proteobacteria(100)	Gammaproteobacter ia(100)	Gammaproteobacteri a_unclassified(100)	Gammaproteobacteria_unclas sified(100)	Wenzhouxiangella marina strain 4S-CH-S3-s2	MG264256
Otu00187	Bacteria(100)	Bacteroidetes(98)	Bacteroidetes_uncla ssified(98)	Bacteroidetes_unclas sified(98)	Bacteroidetes_unclassified(98)	Hydrobacter penzbergensis strain EM 4	NR_134746
Otu00189	Bacteria(100)	Deinococcus-Thermus(100)	Deinococci(100)	Thermales(100)	Thermaceae(100)	Thermus scotoductus SA-01	CP001962
Otu00190	Bacteria(100)	Ignavibacteriae(100)	Ignavibacteria(100)	Ignavibacteriales(100)	Ignavibacteriaceae(100)	Ignavibacterium album strain JCM 16511	NR_074698
Otu00191	Bacteria(100)	Proteobacteria(100)	Alphaproteobacteria (100)	Rhizobiales(100)	Aurantimonadaceae(100)	Aurantimonas aggregata strain	KY984095
Otu00193	Bacteria(100)	Proteobacteria(100)	Deltaproteobacteria (100)	Desulfuromonadales(100)	Desulfuromonadaceae(100)	Desulfuromonas carbonis strain ICBM	KJ776405
Otu00195	Bacteria(100)	Proteobacteria(100)	Gammaproteobacter ia(100)	Vibrionales(100)	Vibrionaceae(100)	Vibrio sp. ER-65	KT325172
Otu00197	Bacteria(100)	Proteobacteria(100)	Proteobacteria_uncl assified(100)	Proteobacteria_uncla ssified(100)	Proteobacteria_unclassified(1 00)	Desulfobacca acetoxidans strain DSM 11109	NR_074955
Otu00198	Bacteria(100)	Proteobacteria(100)	Gammaproteobacter ia(100)	Chromatiales(100)	Chromatiaceae(100)	Allochromatium vinosum DSM 180	CP001896
Otu00199	Bacteria(100)	Proteobacteria(100)	Deltaproteobacteria (92)	Deltaproteobacteria_ unclassified(92)	Deltaproteobacteria_unclassif ied(92)	Anaeromyxobacter sp. PSR-1	AB795400
Otu00200	Bacteria(100)	Firmicutes(100)	Bacilli(100)	Bacillales(100)	Bacillales_unclassified(100)	Paenibacillus solanacearum strain T16R-228	KU379666
Otu00201	Bacteria(100)	Acidobacteria(100)	Acidobacteria_Gp2 3(99)	Gp23(99)	Gp23_unclassified(99)	Thermoanaerobaculum aquaticum strain MP-01	NR_109681
Otu00202	Bacteria(100)	Acidobacteria(100)	Acidobacteria_Gp2 3(100)	Gp23(100)	Nitrososphaera(96)	Candidatus Nitrosotalea devanaterra isolate Nd1	JN227488
Otu00204	Bacteria(100)	Proteobacteria(100)	Betaproteobacteria(100)	Methylophilales(100)	Methylophilaceae(100)	Methylotenera mobilis	AB698738
Otu00205	Bacteria(100)	Firmicutes(100)	Clostridia(100)	Clostridiales(100)	Clostridiales_Incertae_Sedis_ XII(100)	Fusibacter bizertensis strain LTF Kr01	KJ420408
Otu00206	Bacteria(100)	Bacteria_unclassifie d(100)	Bacteria_unclassifie d(100)	Bacteria_unclassified (100)	Bacteria_unclassified(100)	Parcubacteria group bacterium GW2011_GWB1_45_9	KX123379
Otu00208	Bacteria(100)	Bacteria_unclassifie d(100)	Bacteria_unclassifie d(100)	Bacteria_unclassified (100)	Bacteria_unclassified(100)	Polycladomyces subterraneus strain KSR 13	NR_158012

OTU	Taxonomy	Phylum	Class	Order	Family	BLASTn Genus species	Accession
Otu00214	Bacteria(100)	Cyanobacteria/Chloroplast(100)	Cyanobacteria(100)	Family_XIII(100)	GpXIII(100)	Arthrospira platensis strain PCC 7345	NR_125711
Otu00215	Bacteria(100)	Bacteroidetes(100)	Sphingobacteriia(100)	Sphingobacteriales(100)	Rhodothermaceae(100)	Rhodothermus marinus strain R-10	NR_029282
Otu00220	Bacteria(100)	Proteobacteria(100)	Deltaproteobacteria (100)	Syntrophobacterales(100)	Syntrophobacteraceae(100)	Desulfosoma profundi strain SPDX02-08	NR_117786
Otu00223	Bacteria(100)	Thermodesulfobacteria(100)	Thermodesulfobacteria(100)	Thermodesulfobacteriales(100)	Thermodesulfobacteriaceae(100)	Caldimicrobium rimae strain DS	NR_044283
Otu00224	Bacteria(100)	Bacteria_unclassified(100)	Bacteria_unclassified(100)	Bacteria_unclassified (100)	Bacteria_unclassified(100)	Chloroflexi bacterium Ver9Iso1	DQ812549
Otu00225	Bacteria(100)	Bacteroidetes(100)	Sphingobacteriia(100)	Sphingobacteriales(100)	Chitinophagaceae(100)	Terrimonas lutea strain DY	NR_041250
Otu00233	Bacteria(100)	Bacteroidetes(100)	Flavobacteriia(100)	Flavobacteriales(100)	Flavobacteriaceae(100)	Psychroflexus tropicus strain LA1	NR_028854
Otu00234	Bacteria(100)	Proteobacteria(100)	Gammaproteobacteria(100)	Chromatiales(100)	Chromatiaceae(100)	Rheinheimera arenilitoris strain J-MS1	NR_134151
Otu00238	Bacteria(100)	Bacteria_unclassified(100)	Bacteria_unclassified(100)	Bacteria_unclassified (100)	Bacteria_unclassified(100)	Delta proteobacterium TP492	EF636113
Otu00239	Bacteria(100)	Proteobacteria(100)	Betaproteobacteria(95)	Burkholderiales(95)	Burkholderiales_unclassified(89)	Burkholderiales bacterium VC93	FM995606
Otu00246	Bacteria(100)	Bacteria_unclassified(100)	Bacteria_unclassified(100)	Bacteria_unclassified (100)	Bacteria_unclassified(100)	Candidatus Acetothermus autotrophicum	AP011801
Otu00247	Bacteria(100)	Proteobacteria(100)	Alphaproteobacteria (100)	Sphingomonadales(100)	Sphingomonadaceae(100)	Sphingomonadaceae bacterium W45	LC094487
Otu00248	Bacteria(100)	Bacteria_unclassified(100)	Bacteria_unclassified(100)	Bacteria_unclassified (100)	Bacteria_unclassified(100)	Planctomycete GMD14H10 small subunit	AY162122
Otu00252	Bacteria(100)	Acidobacteria(100)	Acidobacteria_Gp16(100)	Gp16(100)	Gp16_unclassified(100)	Bacterium Ellin6529	HM748677
Otu00262	Bacteria(100)	Bacteria_unclassified(100)	Bacteria_unclassified(100)	Bacteria_unclassified (100)	Bacteria_unclassified(100)	Planctomycetes bacterium SCGC AAA001-J07	HQ675380
Otu00267	Bacteria(100)	Proteobacteria(100)	Betaproteobacteria(100)	Neisseriales(100)	Neisseriaceae(100)	Vogesella perlucida strain BN_2063	MG438520
Otu00268	Bacteria(100)	Bacteroidetes(100)	Cytophagia(100)	Cytophagales(100)	Flammeovirgaceae(100)	Roseivirga maritima strain GM-5	KU507204
Otu00271	Bacteria(100)	Bacteroidetes(100)	Flavobacteriia(100)	Flavobacteriales(100)	Flavobacteriaceae(100)	Namhaeicola litoreus strain DPG-25	NR_132282
Otu00282	Bacteria(100)	Proteobacteria(100)	Deltaproteobacteria (100)	Deltaproteobacteria_unclassified(100)	Deltaproteobacteria_unclassified(100)	Chondromyces pediculatus	AJ233940
Otu00285	Bacteria(100)	Bacteria_unclassified(100)	Bacteria_unclassified(100)	Bacteria_unclassified (100)	Bacteria_unclassified(100)	Ammoniphilus oxalivorans strain RAOx-FS	NR_026433

Supplementary Table 6. Statistical summary for non-metric multidimensional scaling (NMDS) and analysis of similarities (ANOSIM) tests

Ordination	NMDS stress	ANOSIM		
		**Classification[a]**	**P value**	**R statistic**
All OTUs	0.14	A	0.001	0.597
		B	0.343	0.039
		C	0.001	0.488
		D	0.001	0.386
		E	0.001	0.543
		F	0.001	0.433
0.1% Abundant OTUs	0.14	A	0.001	0.584
		C	0.001	0.476
		E	0.001	0.533
1% Abundant OTUs	0.14	A	0.001	0.563
		C	0.001	0.468
		E	0.001	0.525
Planktonic Sample OTUs	0.09	A	0.002	0.510
		B	0.662	-0.094
		C	0.004	0.329
		D	0.054	0.229
		E	0.001	0.519
		F	0.001	0.483
Sediment Sample OTUs	0.05	A	0.001	0.694
		B	0.493	-0.017
		C	0.001	0.561
		D	0.003	0.402
		E	0.001	0.544
		F	0.003	0.534
All OTUs excluding Spring 4	0.13	A	0.001	0.268
		C	0.001	0.538
		E	0.001	0.574
1% Abundant OTUs excluding Spring 4	0.14	A	0.005	0.237
		C	0.001	0.516
		E	0.003	0.388
		F	0.001	0.553
1% Abundant OTUs for Springs 1-5	0.04	-	-	-
1% Abundant OTUs for Springs 1-3, 5	0.02	-	-	-
All OTUs for Springs 6-14	0.11	C	0.004	0.532
1% Abundant OTUs for Springs 6-14	0.12	C	0.001	0.533

[a] Classification: A - geochemistry groups based on Principal Component Analysis; B - geochemistry groups based on Piper diagram; C - temperature groups based on microbial temperature preference (i.e. mesophile); D - temperature groups based on 15 ºC increments; E - tectonic setting groups (flat slab and back arc) with Sp. 9 separate; F - tectonic setting groups flat slab springs (FSS) and back arc springs (BAS)

Supplementary Table 7. Partial Mantel test results

Compared matrices[a]		Control matrix[b]	r-stat	significance (p-value)	Upper quantile of permutations[c] (null test), r-statistic			
					90%	95%	97.5%	99%
community 16S rRNA gene OTUs	geochemistry	location	0.3008	0.0099	0.0728	0.0918	0.1213	0.1314
community 16S rRNA gene OTUs	temperature	location	0.2784	0.0099	0.0893	0.1024	0.1138	0.1372
community 16S rRNA gene OTUs	location	temperature	0.1817	0.0099	0.0750	0.0818	0.1085	0.1211
community 16S rRNA gene OTUs	geochemistry	temperature	0.2282	0.0099	0.0690	0.1090	0.1190	0.1260

[a,b] Dissimilarity matrix describing community 16S rRNA gene OTUs (full data set), geochemistry groups from the Principal Component Analysis (PCA), temperature (low, mid, high) groups, and geographic location (flat slab and back arc)
[c] 100 permutations

Supplementary Table 8. Reservoir temperature estimates (RTE) (°C) from Peru flat-slab (FSS) and back-arc (BAS) hot spring geochemistry

Spring	Region	Cation Geothermometry				GeoT	
		Na-K-Ca	Na-K-Ca, Mg corr.	Na-K	K-Mg	no Si[a]	est Si[b]
1	FSS	40	40	245	53	107	66
2	FSS	193	108	269	128	110	96
3	FSS	56	56	144	44	90	81
5	FSS	131	65	242	92	93	105
6	BAS	218	162	282	147	262	256
7	BAS	117	116	253	101	183	248
8	BAS	157	113	231	114	230	240
9	BAS	127	59	195	85	98	126
10	BAS	118	85	186	86	207	135
11	BAS	175	131	188	130	246	252
12	BAS	175	129	189	129	244	223
13	BAS	214	145	218	159	116	116
14	BAS	213	180	240	151	256	124
Mean	FSS	105	67	225	79	100	87
Mean	BAS	168	124	220	122	205	191

[a] RTE determined without automatic Si estimation by iGeoT and without equilibration with silicates
[b] RTE determined with automatic Si estimation by iGeoT and inclusion of silicates
