# Supplementary material for: Tectonic settings influence the geochemical and microbial diversity of Peru hot springs
Source: Commun Earth Environ. 2023 Apr 11;4(1):112. doi: 10.1038/s43247-023-00787-5 (PMC11041657; doi:10.1038/s43247-023-00787-5)
Supplement: Supplementary file 2 — Reporting Summary [file 43247_2023_787_MOESM2_ESM.pdf]

nature portfolio

Corresponding author(s): Dennis L. Newell

Last updated by author(s): Mar 9, 2023

Reporting Summary

Nature Portfolio wishes to improve the reproducibility of the work that we publish. This form provides structure for consistency and transparency in reporting. For further information on Nature Portfolio policies, see our Editorial Policies and the Editorial Policy Checklist.

Statistics

For all statistical analyses, confirm that the following items are present in the figure legend, table legend, main text, or Methods section.

n/a	Confirmed	
☐	☒	The exact sample size (*n*) for each experimental group/condition, given as a discrete number and unit of measurement
☐	☒	A statement on whether measurements were taken from distinct samples or whether the same sample was measured repeatedly
☐	☒	The statistical test(s) used AND whether they are one- or two-sided<br>*Only common tests should be described solely by name; describe more complex techniques in the Methods section.*
☐	☒	A description of all covariates tested
☒	☐	A description of any assumptions or corrections, such as tests of normality and adjustment for multiple comparisons
☐	☒	A full description of the statistical parameters including central tendency (e.g. means) or other basic estimates (e.g. regression coefficient) AND variation (e.g. standard deviation) or associated estimates of uncertainty (e.g. confidence intervals)
☐	☒	For null hypothesis testing, the test statistic (e.g. *F*, *t*, *r*) with confidence intervals, effect sizes, degrees of freedom and *P* value noted<br>*Give P values as exact values whenever suitable.*
☒	☐	For Bayesian analysis, information on the choice of priors and Markov chain Monte Carlo settings
☒	☐	For hierarchical and complex designs, identification of the appropriate level for tests and full reporting of outcomes
☒	☐	Estimates of effect sizes (e.g. Cohen's *d*, Pearson's *r*), indicating how they were calculated

Our web collection on statistics for biologists contains articles on many of the points above.

Software and code

Policy information about availability of computer code

Data collection	no software used for data collection
Data analysis	RStudio (version 1.1.463), Geochemists Workbench version 12.0.4, iGeoT v1.0

For manuscripts utilizing custom algorithms or software that are central to the research but not yet described in published literature, software must be made available to editors and reviewers. We strongly encourage code deposition in a community repository (e.g. GitHub). See the Nature Portfolio guidelines for submitting code & software for further information.

Data

Policy information about availability of data

All manuscripts must include a data availability statement. This statement should provide the following information, where applicable:

- Accession codes, unique identifiers, or web links for publicly available datasets
- A description of any restrictions on data availability
- For clinical datasets or third party data, please ensure that the statement adheres to our policy

Data generated and analyzed in this study are publically available in the Figshare repository: https://doi.org/10.6084/m9.figshare.22233862.v1

Human research participants

Policy information about studies involving human research participants and Sex and Gender in Research.

Reporting on sex and gender	There were no human research participants
Population characteristics	There were no human research participants
Recruitment	There were no human research participants
Ethics oversight	There were no human research participants

Note that full information on the approval of the study protocol must also be provided in the manuscript.

Field-specific reporting

Please select the one below that is the best fit for your research. If you are not sure, read the appropriate sections before making your selection.

☐ Life sciences ☐ Behavioural & social sciences ☒ Ecological, evolutionary & environmental sciences

For a reference copy of the document with all sections, see nature.com/documents/nr-reporting-summary-flat.pdf

Ecological, evolutionary & environmental sciences study design

All studies must disclose on these points even when the disclosure is negative.

Study description	Hot spring grab sampling in Peru. Samples were collected for geochemical and microbiological analyses following published protocols.
Research sample	Research samples in this study comprise water samples collected from hot springs for chemical analyses, and filtered water samples for microbial ecology (i.e., 16S rRNA gene data). Sample locations were based on accessibility and maximum temperature spring emanations if more than one.
Sampling strategy	No statistical methods were used for sampling strategy design. These were grab samples from hot spring emanations, and hot springs were chosen for sampling based on their accessibility.
Data collection	Samples were collected by Dennis Newell and Heather Upin. The two person team collaborated on data entry and sample collection.
Timing and spatial scale	Sampling was conducted over one field season between July 1 and July 10, 2018. Sampling frequency was determined by travel time between locations via vehicle/foot. Some days only one location was sampled, and on others up to 3 locations. The spatial scale is regional ~650 X 300 km.
Data exclusions	no data was excluded
Reproducibility	This was the first time that these hot springs had been characterized using these methods, and the study design did not include goals for reproducibility.
Randomization	The sampling locations were not chosen by randomization. We attempted to collect samples from all of the hot springs present in our study region with exceptions based on inaccessibility or lack of permission from land owners.
Blinding	Blinding is not relevant for hot spring characterization sampling

Did the study involve field work? ☒ Yes ☐ No

Field work, collection and transport

Field conditions	Variable. Generally sunny, occasionally windy, with temperatures ranging from ~ 0 to 20 C
Location	Sampling transect from the southern central highlands (~13°S, 75°W) of Peru to the Altiplano (~15.5°S, 71°W) at elevations of ~3300 to 4700 m above sea level.
Access & import/export	Access and sampling permissions were facilitated through collaborations with Dr. Jose Cardeñas Roque with the Universidad Nacional

Access & import/export	de San Antonio Abad del Cusco in Cusco, Peru. Several hot springs were located at commercial locations, and permission was gained by the property owners. Transportation and shipping of samples followed all applicable laws in Peru and the United States.
Disturbance	no disturbances were caused by this study

Reporting for specific materials, systems and methods

We require information from authors about some types of materials, experimental systems and methods used in many studies. Here, indicate whether each material, system or method listed is relevant to your study. If you are not sure if a list item applies to your research, read the appropriate section before selecting a response.

Materials & experimental systems

n/a	Involved in the study	
☒	☐	Antibodies
☒	☐	Eukaryotic cell lines
☒	☐	Palaeontology and archaeology
☒	☐	Animals and other organisms
☒	☐	Clinical data
☒	☐	Dual use research of concern

Methods

n/a	Involved in the study	
☒	☐	ChIP-seq
☒	☐	Flow cytometry
☒	☐	MRI-based neuroimaging
